# Supplementary material for: Using normative modelling to detect disease progression in mild cognitive impairment and Alzheimer’s disease in a cross-sectional multi-cohort study
Source: Sci Rep. 2021 Aug 3;11:15746. doi: 10.1038/s41598-021-95098-0 (PMC8333350; doi:10.1038/s41598-021-95098-0)
Supplement: Supplementary file 1 — Supplementary Information. [file 41598_2021_95098_MOESM1_ESM.pdf]

# Using normative modelling to detect disease progression in mild cognitive impairment and Alzheimer's disease in a cross-sectional multi-cohort study

Walter H. L. Pinaya [a b c] *, Cristina Scarpazza [a d], Rafael Garcia-Dias [a], Sandra Vieira [a], Lea Baecker [a], Pedro F. da Costa [e f], Alberto Redolfi [g], Giovanni B. Frisoni [h i], Michela Pievani [h], Vince D. Calhoun [j], João R. Sato [b], Andrea Mechelli [a]

* [a] Department of Psychosis Studies, Institute of Psychiatry, Psychology & Neuroscience, King's College London, London, UK.

[b] Center of Mathematics, Computing, and Cognition, Universidade Federal do ABC, Santo André, Brazil.

[c] Department of Biomedical Engineering, School of Biomedical Engineering & Imaging Sciences, King's College London, London, UK

[d] Department of General Psychology, University of Padua, Padua, Italy.

[e] Department of Neuroimaging, Institute of Psychiatry, Psychology & Neuroscience, King's College London, London, UK.

[f] Centre for Brain and Cognitive Development, Birkbeck College, University of London, London, UK.

[g] Laboratory of Neuroinformatics, IRCCS Istituto Centro San Giovanni di Dio Fatebenefratelli, Brescia, Italy.

[h] Laboratory of Alzheimer's Neuroimaging & Epidemiology, IRCCS Istituto Centro San Giovanni di Dio Fatebenefratelli, Brescia, Italy.

[i] Memory Clinic and LANVIE Laboratory of Neuroimaging of Aging, University Hospitals and University of Geneva, Geneva, Switzerland.

[j] Tri-institutional Center for Translational Research in Neuroimaging and Data Science (TReNDS), Georgia State, Georgia Tech, Emory, US

# 1. List of brain features

| | |
|---|---|
| Left-Lateral-Ventricle | lh_parstriangularis_volume |
| Left-Inf-Lat-Vent | lh_pericalcarine_volume |
| Left-Cerebellum-White-Matter | lh_postcentral_volume |
| Left-Cerebellum-Cortex | lh_posteriorcingulate_volume |
| Left-Thalamus-Proper | lh_precentral_volume |
| Left-Caudate | lh_precuneus_volume |
| Left-Putamen | lh_rostralanteriorcingulate_volume |
| Left-Pallidum | lh_rostralmiddlefrontal_volume |
| 3rd-Ventricle | lh_superiorfrontal_volume |
| 4th-Ventricle | lh_superiorparietal_volume |
| Brain-Stem | lh_superiortemporal_volume |
| Left-Hippocampus | lh_supramarginal_volume |
| Left-Amygdala | lh_frontalpole_volume |
| CSF | lh_temporalpole_volume |
| Left-Accumbens-area | lh_transversetemporal_volume |
| Left-VentralDC | lh_insula_volume |
| Right-Lateral-Ventricle | rh_bankssts_volume |
| Right-Inf-Lat-Vent | rh_caudalanteriorcingulate_volume |
| Right-Cerebellum-White-Matter | rh_caudalmiddlefrontal_volume |
| Right-Cerebellum-Cortex | rh_cuneus_volume |
| Right-Thalamus-Proper | rh_entorhinal_volume |
| Right-Caudate | rh_fusiform_volume |
| Right-Putamen | rh_inferiorparietal_volume |
| Right-Pallidum | rh_inferiortemporal_volume |
| Right-Hippocampus | rh_isthmuscingulate_volume |
| Right-Amygdala | rh_lateraloccipital_volume |
| Right-Accumbens-area | rh_lateralorbitofrontal_volume |
| Right-VentralDC | rh_lingual_volume |
| CC_Posterior | rh_medialorbitofrontal_volume |
| CC_Mid_Posterior | rh_middletemporal_volume |
| CC_Central | rh_parahippocampal_volume |
| CC_Mid_Anterior | rh_paracentral_volume |
| CC_Anterior | rh_parsopercularis_volume |
| lh_bankssts_volume | rh_parsorbitalis_volume |
| lh_caudalanteriorcingulate_volume | rh_parstriangularis_volume |
| lh_caudalmiddlefrontal_volume | rh_pericalcarine_volume |
| lh_cuneus_volume | rh_postcentral_volume |
| lh_entorhinal_volume | rh_posteriorcingulate_volume |
| lh_fusiform_volume | rh_precentral_volume |
| lh_inferiorparietal_volume | rh_precuneus_volume |
| lh_inferiortemporal_volume | rh_rostralanteriorcingulate_volume |
| lh_isthmuscingulate_volume | rh_rostralmiddlefrontal_volume |
| lh_lateraloccipital_volume | rh_superiorfrontal_volume |
| lh_lateralorbitofrontal_volume | rh_superiorparietal_volume |
| lh_lingual_volume | rh_superiortemporal_volume |
| lh_medialorbitofrontal_volume | rh_supramarginal_volume |
| lh_middletemporal_volume | rh_frontalpole_volume |
| lh_parahippocampal_volume | rh_temporalpole_volume |
| lh_paracentral_volume | rh_transversetemporal_volume |
| lh_parsopercularis_volume | rh_insula_volume |
| lh_parsorbitalis_volume | |

## 2. Univariate analysis – ADNI dataset– HC vs EMCI

Supplementary Table 1 - Statistical significance measured by the Mann-Whitney U test and effect size measured by Cliff's delta absolute value based on the comparison of the reconstruction error for each brain region between the HC and the EMCI groups from the ADNI dataset. The regions with p-value <= 0.05 are highlighted in bold.

| Regions | Effect size | p-value | Regions | Effect size | p-value |
|---|---|---|---|---|---|
| Left-Lateral-Ventricle | -0.045 | 0.230 | **lh_parstriangularis_volume** | **0.130** | **0.016** |
| **Left-Inf-Lat-Vent** | **-0.112** | **0.033** | lh_pericalcarine_volume | 0.046 | 0.226 |
| Left-Cerebellum-White-Matter | -0.039 | 0.260 | lh_postcentral_volume | 0.077 | 0.101 |
| Left-Cerebellum-Cortex | 0.094 | 0.060 | **lh_posteriorcingulate_volume** | **0.140** | **0.010** |
| **Left-Thalamus-Proper** | **0.209** | **>0.001** | lh_precentral_volume | 0.060 | 0.160 |
| Left-Caudate | 0.062 | 0.153 | **lh_precuneus_volume** | **0.085** | **0.081** |
| Left-Putamen | 0.025 | 0.339 | lh_rostralanteriorcingulate_volume | 0.095 | 0.059 |
| Left-Pallidum | -0.019 | 0.380 | **lh_rostralmiddlefrontal_volume** | **0.124** | **0.021** |
| 3rd-Ventricle | -0.021 | 0.365 | **lh_superiorfrontal_volume** | **0.148** | **0.007** |
| **4th-Ventricle** | **0.153** | **0.006** | lh_superiorparietal_volume | 0.047 | 0.220 |
| Brain-Stem | 0.090 | 0.069 | **lh_superiortemporal_volume** | **0.139** | **0.011** |
| Left-Hippocampus | 0.085 | 0.082 | lh_supramarginal_volume | 0.034 | 0.289 |
| Left-Amygdala | 0.079 | 0.097 | lh_frontalpole_volume | -0.071 | 0.121 |
| **CSF** | **-0.108** | **0.038** | **lh_temporalpole_volume** | **0.143** | **0.009** |
| Left-Accumbens-area | 0.008 | 0.447 | lh_transversetemporal_volume | 0.033 | 0.295 |
| Left-VentralDC | 0.038 | 0.265 | **lh_insula_volume** | **0.189** | **0.001** |
| Right-Lateral-Ventricle | -0.050 | 0.205 | rh_bankssts_volume | 0.005 | 0.466 |
| **Right-Inf-Lat-Vent** | **-0.103** | **0.046** | rh_caudalanteriorcingulate_volume | 0.006 | 0.460 |
| Right-Cerebellum-White-Matter | -0.065 | 0.142 | **rh_caudalmiddlefrontal_volume** | **0.132** | **0.015** |
| **Right-Cerebellum-Cortex** | **0.112** | **0.033** | rh_cuneus_volume | -0.031 | 0.306 |
| **Right-Thalamus-Proper** | **0.199** | **0.001** | rh_entorhinal_volume | 0.010 | 0.437 |
| Right-Caudate | 0.072 | 0.116 | rh_fusiform_volume | 0.062 | 0.153 |
| Right-Putamen | 0.010 | 0.437 | rh_inferiorparietal_volume | 0.065 | 0.141 |
| Right-Pallidum | -0.016 | 0.398 | rh_inferiortemporal_volume | 0.024 | 0.345 |
| **Right-Hippocampus** | **0.123** | **0.022** | rh_isthmuscingulate_volume | 0.068 | 0.130 |
| Right-Amygdala | 0.008 | 0.448 | rh_lateraloccipital_volume | -0.012 | 0.420 |
| **Right-Accumbens-area** | **-0.107** | **0.039** | **rh_lateralorbitofrontal_volume** | **0.091** | **0.067** |
| Right-VentralDC | 0.027 | 0.328 | rh_lingual_volume | -0.050 | 0.207 |
| CC_Posterior | -0.035 | 0.284 | **rh_medialorbitofrontal_volume** | **0.156** | **0.005** |
| CC_Mid_Posterior | 0.029 | 0.318 | **rh_middletemporal_volume** | **0.100** | **0.050** |
| **CC_Central** | **0.103** | **0.045** | rh_parahippocampal_volume | 0.029 | 0.315 |
| CC_Mid_Anterior | 0.031 | 0.304 | **rh_paracentral_volume** | **0.113** | **0.032** |
| CC_Anterior | -0.033 | 0.292 | **rh_parsopercularis_volume** | **0.109** | **0.036** |
| **lh_bankssts_volume** | **0.118** | **0.026** | rh_parsorbitalis_volume | 0.025 | 0.338 |
| lh_caudalanteriorcingulate_volume | 0.065 | 0.140 | rh_parstriangularis_volume | 0.075 | 0.109 |
| **lh_caudalmiddlefrontal_volume** | 0.031 | 0.306 | rh_pericalcarine_volume | 0.078 | 0.099 |
| **lh_cuneus_volume** | -0.041 | 0.248 | **rh_postcentral_volume** | **0.101** | **0.048** |
| **lh_entorhinal_volume** | **-0.092** | **0.065** | rh_posteriorcingulate_volume | 0.056 | 0.179 |
| lh_fusiform_volume | 0.052 | 0.195 | **rh_precentral_volume** | **0.105** | **0.041** |
| **lh_inferiorparietal_volume** | **0.100** | **0.049** | rh_precuneus_volume | 0.042 | 0.242 |
| **lh_inferiortemporal_volume** | **0.092** | **0.064** | **rh_rostralanteriorcingulate_volume** | **0.112** | **0.033** |
| lh_isthmuscingulate_volume | -0.048 | 0.213 | rh_rostralmiddlefrontal_volume | 0.084 | 0.083 |
| lh_lateraloccipital_volume | -0.002 | 0.484 | **rh_superiorfrontal_volume** | **0.114** | **0.030** |
| lh_lateralorbitofrontal_volume | 0.077 | 0.101 | rh_superiorparietal_volume | 0.030 | 0.308 |
| lh_lingual_volume | 0.007 | 0.457 | **rh_superiortemporal_volume** | **0.221** | **0.000** |
| **lh_medialorbitofrontal_volume** | **0.232** | **0.000** | rh_supramarginal_volume | 0.083 | 0.084 |
| **lh_middletemporal_volume** | **0.109** | **0.037** | **rh_frontalpole_volume** | **-0.104** | **0.043** |
| lh_parahippocampal_volume | -0.014 | 0.406 | **rh_temporalpole_volume** | **0.116** | **0.028** |
| **lh_paracentral_volume** | **0.126** | **0.019** | **rh_transversetemporal_volume** | **0.139** | **0.011** |
| **lh_parsopercularis_volume** | **0.134** | **0.014** | **rh_insula_volume** | **0.172** | **0.002** |
| lh_parsorbitalis_volume | -0.012 | 0.424 | | | |

# 3. Univariate analysis – ADNI dataset – HC vs LMCI

Supplementary Table 2 - Statistical significance measured by the Mann-Whitney U test and effect size measured by Cliff's delta absolute value based on the comparison of the reconstruction error for each brain region between the HC and the LMCI groups from the ADNI dataset. The regions with p-value <= 0.05 are highlighted in bold.

| Regions | Effect size | p-value | Regions | Effect size | p-value |
|---|---|---|---|---|---|
| **Left-Lateral-Ventricle** | **-0.106** | **0.080** | **lh_parstriangularis_volume** | **0.232** | **0.001** |
| **Left-Inf-Lat-Vent** | **-0.299** | **>0.001** | lh_pericalcarine_volume | 0.065 | 0.193 |
| Left-Cerebellum-White-Matter | -0.054 | 0.238 | **lh_postcentral_volume** | **0.191** | **0.006** |
| **Left-Cerebellum-Cortex** | **0.127** | **0.045** | **lh_posteriorcingulate_volume** | **0.132** | **0.040** |
| **Left-Thalamus-Proper** | **0.240** | **0.001** | **lh_precentral_volume** | **0.147** | **0.026** |
| Left-Caudate | 0.098 | 0.096 | **lh_precuneus_volume** | **0.246** | **0.001** |
| Left-Putamen | 0.098 | 0.096 | lh_rostralanteriorcingulate_volume | 0.113 | 0.066 |
| Left-Pallidum | 0.016 | 0.415 | **lh_rostralmiddlefrontal_volume** | **0.239** | **0.001** |
| **3rd-Ventricle** | **-0.161** | **0.016** | **lh_superiorfrontal_volume** | **0.287** | **>0.001** |
| 4th-Ventricle | 0.039 | 0.301 | lh_superiorparietal_volume | 0.120 | 0.055 |
| Brain-Stem | 0.068 | 0.183 | **lh_superiortemporal_volume** | **0.298** | **>0.001** |
| **Left-Hippocampus** | **0.366** | **>0.001** | **lh_supramarginal_volume** | **0.231** | **0.001** |
| **Left-Amygdala** | **0.278** | **>0.001** | lh_frontalpole_volume | 0.123 | 0.051 |
| **CSF** | **-0.245** | **0.001** | **lh_temporalpole_volume** | **0.142** | **0.029** |
| **Left-Accumbens-area** | **0.222** | **0.002** | lh_transversetemporal_volume | 0.086 | 0.126 |
| Left-VentralDC | 0.112 | 0.068 | **lh_insula_volume** | **0.254** | **>0.001** |
| Right-Lateral-Ventricle | -0.106 | 0.079 | **rh_bankssts_volume** | **0.227** | **0.001** |
| **Right-Inf-Lat-Vent** | **-0.258** | **>0.001** | rh_caudalanteriorcingulate_volume | 0.001 | 0.493 |
| Right-Cerebellum-White-Matter | -0.108 | 0.076 | **rh_caudalmiddlefrontal_volume** | **0.142** | **0.029** |
| Right-Cerebellum-Cortex | 0.118 | 0.058 | rh_cuneus_volume | 0.079 | 0.147 |
| **Right-Thalamus-Proper** | **0.336** | **>0.001** | **rh_entorhinal_volume** | **0.214** | **0.002** |
| Right-Caudate | 0.066 | 0.192 | **rh_fusiform_volume** | **0.198** | **0.004** |
| Right-Putamen | 0.087 | 0.125 | **rh_inferiorparietal_volume** | **0.345** | **>0.001** |
| Right-Pallidum | -0.044 | 0.280 | **rh_inferiortemporal_volume** | **0.267** | **>0.001** |
| **Right-Hippocampus** | **0.382** | **>0.001** | rh_isthmuscingulate_volume | 0.106 | 0.080 |
| **Right-Amygdala** | **0.157** | **0.018** | **rh_lateraloccipital_volume** | **0.173** | **0.011** |
| Right-Accumbens-area | 0.026 | 0.363 | **rh_lateralorbitofrontal_volume** | **0.125** | **0.048** |
| Right-VentralDC | 0.106 | 0.080 | rh_lingual_volume | 0.081 | 0.141 |
| CC_Posterior | 0.013 | 0.429 | **rh_medialorbitofrontal_volume** | **0.189** | **0.006** |
| CC_Mid_Posterior | -0.032 | 0.337 | **rh_middletemporal_volume** | **0.349** | **>0.001** |
| CC_Central | 0.083 | 0.134 | **rh_parahippocampal_volume** | **0.178** | **0.009** |
| CC_Mid_Anterior | 0.105 | 0.081 | **rh_paracentral_volume** | **0.167** | **0.013** |
| CC_Anterior | 0.012 | 0.436 | **rh_parsopercularis_volume** | **0.126** | **0.047** |
| **lh_bankssts_volume** | **0.335** | **>0.001** | **rh_parsorbitalis_volume** | **0.139** | **0.032** |
| lh_caudalanteriorcingulate_volume | -0.092 | 0.110 | **rh_parstriangularis_volume** | **0.212** | **0.002** |
| **lh_caudalmiddlefrontal_volume** | **0.240** | **0.001** | rh_pericalcarine_volume | 0.052 | 0.245 |
| lh_cuneus_volume | 0.012 | 0.435 | rh_postcentral_volume | 0.089 | 0.120 |
| **lh_entorhinal_volume** | **0.210** | **0.003** | **rh_posteriorcingulate_volume** | **0.265** | **>0.001** |
| **lh_fusiform_volume** | **0.371** | **>0.001** | rh_precentral_volume | 0.034 | 0.327 |
| **lh_inferiorparietal_volume** | **0.377** | **>0.001** | **rh_precuneus_volume** | **0.237** | **0.001** |
| **lh_inferiortemporal_volume** | **0.227** | **0.001** | rh_rostralanteriorcingulate_volume | 0.057 | 0.226 |
| **lh_isthmuscingulate_volume** | **0.130** | **0.043** | **rh_rostralmiddlefrontal_volume** | **0.271** | **>0.001** |
| lh_lateraloccipital_volume | 0.122 | 0.053 | **rh_superiorfrontal_volume** | **0.249** | **>0.001** |
| **lh_lateralorbitofrontal_volume** | **0.145** | **0.027** | **rh_superiorparietal_volume** | **0.241** | **0.001** |
| lh_lingual_volume | 0.112 | 0.069 | **rh_superiortemporal_volume** | **0.334** | **>0.001** |
| **lh_medialorbitofrontal_volume** | **0.197** | **0.004** | **rh_supramarginal_volume** | **0.230** | **0.001** |
| **lh_middletemporal_volume** | **0.344** | **>0.001** | rh_frontalpole_volume | -0.029 | 0.352 |
| **lh_parahippocampal_volume** | **0.167** | **0.013** | rh_temporalpole_volume | 0.089 | 0.118 |
| **lh_paracentral_volume** | **0.125** | **0.048** | rh_transversetemporal_volume | 0.033 | 0.332 |
| **lh_parsopercularis_volume** | **0.247** | **0.001** | **rh_insula_volume** | **0.204** | **0.003** |
| lh_parsorbitalis_volume | 0.075 | 0.160 | | | |

## 4. Univariate analysis – ADNI dataset – HC vs AD

Supplementary Table 3 - Statistical significance measured by the Mann-Whitney U test and effect size measured by Cliff's delta absolute value based on the comparison of the reconstruction error for each brain region between the HC and the AD groups from the ADNI dataset. The regions with p-value <= 0.05 are highlighted in bold.

| Regions | Effect size | p-value | Regions | Effect size | p-value |
|---|---|---|---|---|---|
| **Left-Lateral-Ventricle** | **-0.552** | **>0.001** | **lh_parstriangularis_volume** | **0.420** | **>0.001** |
| **Left-Inf-Lat-Vent** | **-0.708** | **>0.001** | **lh_pericalcarine_volume** | **0.246** | **0.001** |
| Left-Cerebellum-White-Matter | -0.040 | 0.316 | **lh_postcentral_volume** | **0.330** | **>0.001** |
| Left-Cerebellum-Cortex | 0.103 | 0.106 | **lh_posteriorcingulate_volume** | **0.414** | **>0.001** |
| **Left-Thalamus-Proper** | **0.488** | **>0.001** | **lh_precentral_volume** | **0.317** | **>0.001** |
| **Left-Caudate** | **0.179** | **0.015** | **lh_precuneus_volume** | **0.640** | **>0.001** |
| **Left-Putamen** | **0.378** | **>0.001** | **lh_rostralanteriorcingulate_volume** | **0.328** | **>0.001** |
| Left-Pallidum | -0.052 | 0.264 | **lh_rostralmiddlefrontal_volume** | **0.581** | **>0.001** |
| **3rd-Ventricle** | **-0.542** | **>0.001** | **lh_superiorfrontal_volume** | **0.633** | **>0.001** |
| 4th-Ventricle | -0.075 | 0.181 | **lh_superiorparietal_volume** | **0.424** | **>0.001** |
| **Brain-Stem** | **0.189** | **0.011** | **lh_superiortemporal_volume** | **0.676** | **>0.001** |
| **Left-Hippocampus** | **0.748** | **>0.001** | **lh_supramarginal_volume** | **0.587** | **>0.001** |
| **Left-Amygdala** | **0.768** | **>0.001** | **lh_frontalpole_volume** | **0.204** | **0.007** |
| **CSF** | **-0.528** | **>0.001** | **lh_temporalpole_volume** | **0.203** | **0.007** |
| **Left-Accumbens-area** | **0.458** | **>0.001** | **lh_transversetemporal_volume** | **0.276** | **>0.001** |
| **Left-VentralDC** | **0.277** | **>0.001** | **lh_insula_volume** | **0.401** | **>0.001** |
| **Right-Lateral-Ventricle** | **-0.533** | **>0.001** | **rh_bankssts_volume** | **0.554** | **>0.001** |
| **Right-Inf-Lat-Vent** | **-0.705** | **>0.001** | rh_caudalanteriorcingulate_volume | 0.082 | 0.160 |
| Right-Cerebellum-White-Matter | -0.080 | 0.166 | **rh_caudalmiddlefrontal_volume** | **0.442** | **>0.001** |
| **Right-Cerebellum-Cortex** | **0.153** | **0.032** | **rh_cuneus_volume** | **0.148** | **0.037** |
| **Right-Thalamus-Proper** | **0.499** | **>0.001** | **rh_entorhinal_volume** | **0.430** | **>0.001** |
| **Right-Caudate** | **0.136** | **0.049** | **rh_fusiform_volume** | **0.698** | **>0.001** |
| **Right-Putamen** | **0.371** | **>0.001** | **rh_inferiorparietal_volume** | **0.618** | **>0.001** |
| Right-Pallidum | 0.002 | 0.491 | **rh_inferiortemporal_volume** | **0.621** | **>0.001** |
| **Right-Hippocampus** | **0.719** | **>0.001** | **rh_isthmuscingulate_volume** | **0.412** | **>0.001** |
| **Right-Amygdala** | **0.685** | **>0.001** | **rh_lateraloccipital_volume** | **0.422** | **>0.001** |
| **Right-Accumbens-area** | **0.349** | **>0.001** | **rh_lateralorbitofrontal_volume** | **0.422** | **>0.001** |
| **Right-VentralDC** | **0.230** | **0.003** | **rh_lingual_volume** | **0.231** | **0.003** |
| CC_Posterior | -0.026 | 0.376 | **rh_medialorbitofrontal_volume** | **0.415** | **>0.001** |
| **CC_Mid_Posterior** | **0.177** | **0.016** | **rh_middletemporal_volume** | **0.697** | **>0.001** |
| **CC_Central** | **0.383** | **>0.001** | **rh_parahippocampal_volume** | **0.416** | **>0.001** |
| **CC_Mid_Anterior** | **0.306** | **>0.001** | **rh_paracentral_volume** | **0.264** | **0.001** |
| CC_Anterior | 0.059 | 0.238 | **rh_parsopercularis_volume** | **0.310** | **>0.001** |
| **lh_bankssts_volume** | **0.656** | **>0.001** | **rh_parsorbitalis_volume** | **0.349** | **>0.001** |
| lh_caudalanteriorcingulate_volume | -0.035 | 0.337 | **rh_parstriangularis_volume** | **0.266** | **0.001** |
| **lh_caudalmiddlefrontal_volume** | **0.487** | **>0.001** | **rh_pericalcarine_volume** | **0.211** | **0.005** |
| **lh_cuneus_volume** | **0.200** | **0.008** | **rh_postcentral_volume** | **0.318** | **>0.001** |
| **lh_entorhinal_volume** | **0.514** | **>0.001** | **rh_posteriorcingulate_volume** | **0.427** | **>0.001** |
| **lh_fusiform_volume** | **0.621** | **>0.001** | **rh_precentral_volume** | **0.280** | **>0.001** |
| **lh_inferiorparietal_volume** | **0.644** | **>0.001** | **rh_precuneus_volume** | **0.573** | **>0.001** |
| **lh_inferiortemporal_volume** | **0.641** | **>0.001** | **rh_rostralanteriorcingulate_volume** | **0.143** | **0.041** |
| **lh_isthmuscingulate_volume** | **0.446** | **>0.001** | **rh_rostralmiddlefrontal_volume** | **0.555** | **>0.001** |
| **lh_lateraloccipital_volume** | **0.388** | **>0.001** | **rh_superiorfrontal_volume** | **0.450** | **>0.001** |
| **lh_lateralorbitofrontal_volume** | **0.439** | **>0.001** | **rh_superiorparietal_volume** | **0.519** | **>0.001** |
| **lh_lingual_volume** | **0.354** | **>0.001** | **rh_superiortemporal_volume** | **0.613** | **>0.001** |
| **lh_medialorbitofrontal_volume** | **0.453** | **>0.001** | **rh_supramarginal_volume** | **0.572** | **>0.001** |
| **lh_middletemporal_volume** | **0.686** | **>0.001** | rh_frontalpole_volume | 0.072 | 0.190 |
| **lh_parahippocampal_volume** | **0.458** | **>0.001** | **rh_temporalpole_volume** | **0.297** | **>0.001** |
| **lh_paracentral_volume** | **0.250** | **0.001** | **rh_transversetemporal_volume** | **0.230** | **0.003** |
| **lh_parsopercularis_volume** | **0.382** | **>0.001** | **rh_insula_volume** | **0.468** | **>0.001** |
| **lh_parsorbitalis_volume** | **0.271** | **0.001** | | | |

## 5. Univariate analysis – AIBL dataset – HC vs MCI

Supplementary Table 4 - Statistical significance measured by the Mann-Whitney U test and effect size measured by Cliff's delta absolute value based on the comparison of the reconstruction error for each brain region between the HC and the MCI groups from the AIBL dataset. The regions with p-value <= 0.05 are highlighted in bold.

| Regions | Effect size | p-value | Regions | Effect size | p-value |
|---|---|---|---|---|---|
| Left-Lateral-Ventricle | -0.125 | 0.088 | lh_parstriangularis_volume | -0.008 | 0.466 |
| **Left-Inf-Lat-Vent** | **-0.273** | **0.002** | lh_pericalcarine_volume | 0.109 | 0.120 |
| Left-Cerebellum-White-Matter | 0.128 | 0.084 | lh_postcentral_volume | 0.144 | 0.059 |
| Left-Cerebellum-Cortex | 0.140 | 0.065 | lh_posteriorcingulate_volume | 0.075 | 0.210 |
| Left-Thalamus-Proper | 0.104 | 0.132 | lh_precentral_volume | 0.067 | 0.236 |
| Left-Caudate | 0.115 | 0.106 | **lh_precuneus_volume** | **0.294** | **0.001** |
| Left-Putamen | -0.013 | 0.444 | lh_rostralanteriorcingulate_volume | 0.090 | 0.164 |
| Left-Pallidum | -0.045 | 0.313 | **lh_rostralmiddlefrontal_volume** | **0.205** | **0.013** |
| **3rd-Ventricle** | **-0.234** | **0.006** | **lh_superiorfrontal_volume** | **0.236** | **0.005** |
| 4th-Ventricle | -0.037 | 0.343 | lh_superiorparietal_volume | 0.077 | 0.202 |
| Brain-Stem | 0.005 | 0.480 | **lh_superiortemporal_volume** | **0.261** | **0.002** |
| **Left-Hippocampus** | **0.299** | **0.001** | lh_supramarginal_volume | 0.105 | 0.129 |
| **Left-Amygdala** | **0.218** | **0.009** | lh_frontalpole_volume | 0.116 | 0.106 |
| **CSF** | **-0.323** | **0.000** | **lh_temporalpole_volume** | **0.258** | **0.003** |
| Left-Accumbens-area | -0.019 | 0.417 | lh_transversetemporal_volume | 0.038 | 0.341 |
| Left-VentralDC | -0.017 | 0.427 | **lh_insula_volume** | **0.223** | **0.008** |
| **Right-Lateral-Ventricle** | **-0.167** | **0.035** | rh_bankssts_volume | 0.108 | 0.123 |
| **Right-Inf-Lat-Vent** | **-0.364** | **>0.001** | rh_caudalanteriorcingulate_volume | 0.000 | 0.500 |
| Right-Cerebellum-White-Matter | 0.145 | 0.058 | rh_caudalmiddlefrontal_volume | 0.150 | 0.053 |
| **Right-Cerebellum-Cortex** | **0.159** | **0.042** | rh_cuneus_volume | 0.108 | 0.122 |
| **Right-Thalamus-Proper** | **0.165** | **0.037** | rh_entorhinal_volume | 0.150 | 0.052 |
| Right-Caudate | 0.029 | 0.379 | **rh_fusiform_volume** | **0.243** | **0.004** |
| Right-Putamen | -0.001 | 0.496 | **rh_inferiorparietal_volume** | **0.173** | **0.031** |
| Right-Pallidum | 0.020 | 0.413 | rh_inferiortemporal_volume | 0.140 | 0.065 |
| **Right-Hippocampus** | **0.311** | **>0.001** | **rh_isthmuscingulate_volume** | **0.260** | **0.003** |
| **Right-Amygdala** | **0.210** | **0.012** | rh_lateraloccipital_volume | 0.129 | 0.082 |
| Right-Accumbens-area | -0.025 | 0.392 | **rh_lateralorbitofrontal_volume** | **0.253** | **0.003** |
| Right-VentralDC | -0.077 | 0.201 | **rh_lingual_volume** | **0.189** | **0.020** |
| CC_Posterior | 0.008 | 0.467 | rh_medialorbitofrontal_volume | 0.115 | 0.106 |
| CC_Mid_Posterior | 0.117 | 0.104 | **rh_middletemporal_volume** | **0.176** | **0.029** |
| CC_Central | 0.048 | 0.302 | **rh_parahippocampal_volume** | **0.154** | **0.048** |
| CC_Mid_Anterior | 0.118 | 0.101 | rh_paracentral_volume | 0.134 | 0.073 |
| CC_Anterior | 0.062 | 0.250 | rh_parsopercularis_volume | 0.106 | 0.126 |
| **lh_bankssts_volume** | **0.203** | **0.014** | rh_parsorbitalis_volume | 0.055 | 0.278 |
| lh_caudalanteriorcingulate_volume | 0.030 | 0.374 | rh_parstriangularis_volume | 0.093 | 0.158 |
| **lh_caudalmiddlefrontal_volume** | **0.189** | **0.021** | rh_pericalcarine_volume | 0.077 | 0.203 |
| **lh_cuneus_volume** | **0.193** | **0.018** | **rh_postcentral_volume** | **0.173** | **0.031** |
| **lh_entorhinal_volume** | **0.340** | **>0.001** | rh_posteriorcingulate_volume | 0.063 | 0.247 |
| **lh_fusiform_volume** | **0.249** | **0.003** | rh_precentral_volume | 0.131 | 0.079 |
| lh_inferiorparietal_volume | 0.089 | 0.168 | **rh_precuneus_volume** | **0.223** | **0.008** |
| lh_inferiortemporal_volume | 0.143 | 0.061 | **rh_rostralanteriorcingulate_volume** | **0.177** | **0.028** |
| lh_isthmuscingulate_volume | 0.136 | 0.071 | rh_rostralmiddlefrontal_volume | 0.091 | 0.162 |
| lh_lateraloccipital_volume | 0.142 | 0.062 | **rh_superiorfrontal_volume** | **0.250** | **0.003** |
| **lh_lateralorbitofrontal_volume** | **0.248** | **0.004** | **rh_superiorparietal_volume** | **0.241** | **0.005** |
| lh_lingual_volume | 0.126 | 0.087 | **rh_superiortemporal_volume** | **0.239** | **0.005** |
| **lh_medialorbitofrontal_volume** | **0.292** | **0.001** | rh_supramarginal_volume | 0.092 | 0.160 |
| lh_middletemporal_volume | 0.120 | 0.097 | rh_frontalpole_volume | 0.081 | 0.189 |
| lh_parahippocampal_volume | 0.095 | 0.151 | **rh_temporalpole_volume** | **0.188** | **0.021** |
| lh_paracentral_volume | 0.101 | 0.138 | **rh_transversetemporal_volume** | **0.197** | **0.017** |
| lh_parsopercularis_volume | 0.049 | 0.298 | **rh_insula_volume** | **0.252** | **0.003** |
| lh_parsorbitalis_volume | 0.038 | 0.341 | | | |

# 6. Univariate analysis – AIBL dataset – HC vs AD

Supplementary Table 5 - Statistical significance measured by the Mann-Whitney U test and effect size measured by Cliff's delta absolute value based on the comparison of the reconstruction error for each brain region between the HC and the AD groups from AIBL dataset. The regions with p-value <= 0.05 are highlighted in bold.

| Regions | Effect size | p-value | Regions | Effect size | p-value |
|---|---|---|---|---|---|
| **Left-Lateral-Ventricle** | **-0.584** | **>0.001** | **lh_parstriangularis_volume** | **0.275** | **0.003** |
| **Left-Inf-Lat-Vent** | **-0.803** | **>0.001** | lh_pericalcarine_volume | 0.133 | 0.092 |
| **Left-Cerebellum-White-Matter** | **0.224** | **0.013** | lh_postcentral_volume | 0.137 | 0.086 |
| **Left-Cerebellum-Cortex** | **0.307** | **0.001** | **lh_posteriorcingulate_volume** | **0.416** | **0.000** |
| **Left-Thalamus-Proper** | **0.335** | **>0.001** | lh_precentral_volume | 0.075 | 0.228 |
| Left-Caudate | 0.147 | 0.072 | **lh_precuneus_volume** | **0.721** | **>0.001** |
| **Left-Putamen** | **0.284** | **0.002** | **lh_rostralanteriorcingulate_volume** | **0.387** | **>0.001** |
| Left-Pallidum | 0.151 | 0.066 | **lh_rostralmiddlefrontal_volume** | **0.510** | **>0.001** |
| **3rd-Ventricle** | **-0.510** | **>0.001** | **lh_superiorfrontal_volume** | **0.326** | **0.001** |
| 4th-Ventricle | -0.079 | 0.216 | **lh_superiorparietal_volume** | **0.447** | **>0.001** |
| **Brain-Stem** | **0.197** | **0.025** | **lh_superiortemporal_volume** | **0.481** | **>0.001** |
| **Left-Hippocampus** | **0.729** | **>0.001** | **lh_supramarginal_volume** | **0.382** | **>0.001** |
| **Left-Amygdala** | **0.666** | **>0.001** | **lh_frontalpole_volume** | **0.184** | **0.034** |
| **CSF** | **-0.492** | **>0.001** | **lh_temporalpole_volume** | **0.238** | **0.009** |
| **Left-Accumbens-area** | **0.217** | **0.015** | lh_transversetemporal_volume | 0.113 | 0.130 |
| **Left-VentralDC** | **0.226** | **0.012** | **lh_insula_volume** | **0.272** | **0.003** |
| **Right-Lateral-Ventricle** | **-0.610** | **>0.001** | **rh_bankssts_volume** | **0.540** | **0.000** |
| **Right-Inf-Lat-Vent** | **-0.764** | **>0.001** | **rh_caudalanteriorcingulate_volume** | **0.241** | **0.008** |
| **Right-Cerebellum-White-Matter** | **0.203** | **0.022** | **rh_caudalmiddlefrontal_volume** | **0.386** | **>0.001** |
| **Right-Cerebellum-Cortex** | **0.313** | **0.001** | **rh_cuneus_volume** | **0.287** | **0.002** |
| **Right-Thalamus-Proper** | **0.356** | **>0.001** | **rh_entorhinal_volume** | **0.734** | **>0.001** |
| Right-Caudate | 0.151 | 0.067 | **rh_fusiform_volume** | **0.533** | **>0.001** |
| **Right-Putamen** | **0.276** | **0.003** | **rh_inferiorparietal_volume** | **0.728** | **>0.001** |
| Right-Pallidum | 0.141 | 0.080 | **rh_inferiortemporal_volume** | **0.720** | **>0.001** |
| **Right-Hippocampus** | **0.751** | **>0.001** | **rh_isthmuscingulate_volume** | **0.595** | **>0.001** |
| **Right-Amygdala** | **0.737** | **>0.001** | **rh_lateraloccipital_volume** | **0.485** | **>0.001** |
| **Right-Accumbens-area** | **0.362** | **>0.001** | **rh_lateralorbitofrontal_volume** | **0.407** | **>0.001** |
| **Right-VentralDC** | **0.270** | **0.004** | **rh_lingual_volume** | **0.339** | **>0.001** |
| CC_Posterior | 0.070 | 0.244 | **rh_medialorbitofrontal_volume** | **0.340** | **>0.001** |
| **CC_Mid_Posterior** | **0.307** | **0.001** | **rh_middletemporal_volume** | **0.609** | **>0.001** |
| **CC_Central** | **0.393** | **>0.001** | **rh_parahippocampal_volume** | **0.576** | **>0.001** |
| **CC_Mid_Anterior** | **0.427** | **>0.001** | rh_paracentral_volume | 0.103 | 0.153 |
| CC_Anterior | 0.084 | 0.203 | **rh_parsopercularis_volume** | **0.305** | **0.001** |
| **lh_bankssts_volume** | **0.389** | **>0.001** | **rh_parsorbitalis_volume** | **0.326** | **0.001** |
| lh_caudalanteriorcingulate_volume | 0.045 | 0.326 | **rh_parstriangularis_volume** | **0.309** | **0.001** |
| **lh_caudalmiddlefrontal_volume** | **0.312** | **0.001** | rh_pericalcarine_volume | 0.161 | 0.054 |
| **lh_cuneus_volume** | **0.357** | **>0.001** | rh_postcentral_volume | 0.046 | 0.322 |
| **lh_entorhinal_volume** | **0.719** | **>0.001** | **rh_posteriorcingulate_volume** | **0.360** | **>0.001** |
| **lh_fusiform_volume** | **0.586** | **>0.001** | rh_precentral_volume | 0.074 | 0.231 |
| **lh_inferiorparietal_volume** | **0.636** | **>0.001** | **rh_precuneus_volume** | **0.652** | **>0.001** |
| **lh_inferiortemporal_volume** | **0.585** | **>0.001** | **rh_rostralanteriorcingulate_volume** | **0.388** | **>0.001** |
| **lh_isthmuscingulate_volume** | **0.590** | **>0.001** | **rh_rostralmiddlefrontal_volume** | **0.469** | **>0.001** |
| **lh_lateraloccipital_volume** | **0.589** | **>0.001** | **rh_superiorfrontal_volume** | **0.304** | **0.001** |
| **lh_lateralorbitofrontal_volume** | **0.407** | **>0.001** | **rh_superiorparietal_volume** | **0.596** | **>0.001** |
| **lh_lingual_volume** | **0.283** | **0.002** | **rh_superiortemporal_volume** | **0.441** | **>0.001** |
| **lh_medialorbitofrontal_volume** | **0.214** | **0.016** | **rh_supramarginal_volume** | **0.496** | **>0.001** |
| **lh_middletemporal_volume** | **0.624** | **>0.001** | rh_frontalpole_volume | 0.092 | 0.179 |
| **lh_parahippocampal_volume** | **0.485** | **>0.001** | **rh_temporalpole_volume** | **0.381** | **>0.001** |
| lh_paracentral_volume | 0.137 | 0.087 | **rh_transversetemporal_volume** | **0.219** | **0.014** |
| **lh_parsopercularis_volume** | **0.365** | **>0.001** | **rh_insula_volume** | **0.439** | **>0.001** |
| **lh_parsorbitalis_volume** | **0.256** | **0.005** | | | |

# 7. Univariate analysis – ARWIBO dataset – HC vs MCI

Supplementary Table 6 - Statistical significance measured by the Mann-Whitney U test and effect size measured by Cliff's delta absolute value based on the comparison of the reconstruction error for each brain region between the HC and the MCI groups from the ARWIBO dataset. The regions with p-value <= 0.05 are highlighted in bold.

| Regions | Effect size | p-value | Regions | Effect size | p-value |
|---|---|---|---|---|---|
| **Left-Lateral-Ventricle** | **-0.251** | **0.001** | **lh_parstriangularis_volume** | **0.199** | **0.007** |
| **Left-Inf-Lat-Vent** | **-0.250** | **0.001** | lh_pericalcarine_volume | -0.018 | 0.412 |
| **Left-Cerebellum-White-Matter** | **0.167** | **0.020** | lh_postcentral_volume | 0.043 | 0.296 |
| Left-Cerebellum-Cortex | -0.085 | 0.147 | **lh_posteriorcingulate_volume** | **0.236** | **0.002** |
| **Left-Thalamus-Proper** | **0.210** | **0.005** | **lh_precentral_volume** | **0.145** | **0.036** |
| Left-Caudate | 0.047 | 0.280 | **lh_precuneus_volume** | **0.229** | **0.002** |
| Left-Putamen | 0.084 | 0.149 | **lh_rostralanteriorcingulate_volume** | **0.139** | **0.043** |
| Left-Pallidum | 0.010 | 0.452 | **lh_rostralmiddlefrontal_volume** | **0.189** | **0.010** |
| **3rd-Ventricle** | **-0.235** | **0.002** | **lh_superiorfrontal_volume** | **0.226** | **0.003** |
| **4th-Ventricle** | **-0.170** | **0.018** | lh_superiorparietal_volume | 0.132 | 0.051 |
| **Brain-Stem** | **0.148** | **0.034** | lh_superiortemporal_volume | -0.020 | 0.402 |
| **Left-Hippocampus** | **0.276** | **>0.001** | lh_supramarginal_volume | 0.070 | 0.193 |
| Left-Amygdala | 0.122 | 0.066 | lh_frontalpole_volume | -0.037 | 0.323 |
| **CSF** | **-0.285** | **>0.001** | lh_temporalpole_volume | 0.071 | 0.190 |
| **Left-Accumbens-area** | **0.277** | **>0.001** | lh_transversetemporal_volume | -0.132 | 0.051 |
| Left-VentralDC | 0.119 | 0.071 | **lh_insula_volume** | **0.185** | **0.011** |
| **Right-Lateral-Ventricle** | **-0.261** | **0.001** | **rh_bankssts_volume** | **0.247** | **0.001** |
| **Right-Inf-Lat-Vent** | **-0.335** | **>0.001** | rh_caudalanteriorcingulate_volume | 0.039 | 0.315 |
| Right-Cerebellum-White-Matter | 0.037 | 0.325 | **rh_caudalmiddlefrontal_volume** | **0.243** | **0.001** |
| Right-Cerebellum-Cortex | -0.048 | 0.276 | rh_cuneus_volume | -0.026 | 0.376 |
| **Right-Thalamus-Proper** | **0.170** | **0.018** | **rh_entorhinal_volume** | **0.272** | **>0.001** |
| Right-Caudate | 0.110 | 0.087 | **rh_fusiform_volume** | **0.306** | **>0.001** |
| Right-Putamen | 0.095 | 0.121 | **rh_inferiorparietal_volume** | **0.271** | **>0.001** |
| Right-Pallidum | 0.085 | 0.148 | **rh_inferiortemporal_volume** | **0.344** | **>0.001** |
| **Right-Hippocampus** | **0.269** | **>0.001** | **rh_isthmuscingulate_volume** | **0.137** | **0.045** |
| **Right-Amygdala** | **0.237** | **0.002** | **rh_lateraloccipital_volume** | **0.135** | **0.048** |
| Right-Accumbens-area | 0.109 | 0.088 | **rh_lateralorbitofrontal_volume** | **0.195** | **0.008** |
| Right-VentralDC | 0.106 | 0.095 | **rh_lingual_volume** | **0.165** | **0.021** |
| CC_Posterior | 0.110 | 0.088 | **rh_medialorbitofrontal_volume** | **0.232** | **0.002** |
| CC_Mid_Posterior | 0.120 | 0.069 | **rh_middletemporal_volume** | **0.228** | **0.002** |
| **CC_Central** | **0.147** | **0.035** | **rh_parahippocampal_volume** | **0.228** | **0.002** |
| CC_Mid_Anterior | 0.089 | 0.135 | **rh_paracentral_volume** | **0.166** | **0.020** |
| CC_Anterior | 0.068 | 0.202 | rh_parsopercularis_volume | 0.098 | 0.114 |
| lh_bankssts_volume | 0.085 | 0.146 | **rh_parsorbitalis_volume** | **0.181** | **0.013** |
| lh_caudalanteriorcingulate_volume | 0.125 | 0.062 | **rh_parstriangularis_volume** | **0.168** | **0.019** |
| **lh_caudalmiddlefrontal_volume** | **0.147** | **0.035** | rh_pericalcarine_volume | 0.093 | 0.126 |
| lh_cuneus_volume | -0.025 | 0.378 | rh_postcentral_volume | -0.020 | 0.400 |
| lh_entorhinal_volume | 0.203 | 0.006 | **rh_posteriorcingulate_volume** | **0.244** | **0.001** |
| **lh_fusiform_volume** | **0.262** | **0.001** | **rh_precentral_volume** | **0.198** | **0.007** |
| lh_inferiorparietal_volume | 0.198 | 0.007 | **rh_precuneus_volume** | **0.256** | **0.001** |
| **lh_inferiortemporal_volume** | **0.275** | **>0.001** | **rh_rostralanteriorcingulate_volume** | **0.191** | **0.009** |
| **lh_isthmuscingulate_volume** | **0.155** | **0.028** | rh_rostralmiddlefrontal_volume | 0.119 | 0.070 |
| **lh_lateraloccipital_volume** | **0.176** | **0.015** | **rh_superiorfrontal_volume** | **0.261** | **0.001** |
| **lh_lateralorbitofrontal_volume** | **0.193** | **0.009** | **rh_superiorparietal_volume** | **0.204** | **0.006** |
| **lh_lingual_volume** | **0.175** | **0.015** | rh_superiortemporal_volume | 0.097 | 0.117 |
| **lh_medialorbitofrontal_volume** | **0.253** | **0.001** | rh_supramarginal_volume | 0.131 | 0.053 |
| **lh_middletemporal_volume** | **0.280** | **>0.001** | rh_frontalpole_volume | -0.023 | 0.389 |
| **lh_parahippocampal_volume** | **0.325** | **>0.001** | rh_temporalpole_volume | 0.084 | 0.150 |
| lh_paracentral_volume | 0.086 | 0.145 | rh_transversetemporal_volume | 0.048 | 0.276 |
| **lh_parsopercularis_volume** | **0.163** | **0.022** | **rh_insula_volume** | **0.149** | **0.033** |
| **lh_parsorbitalis_volume** | **0.209** | **0.005** | | | |

# 8. Univariate analysis – ARWIBO dataset – HC vs AD

Supplementary Table 7 - Statistical significance measured by the Mann-Whitney U test and effect size measured by Cliff's delta absolute value based on the comparison of the reconstruction error for each brain region between the HC and the AD groups from ARWIBO dataset. The regions with p-value <= 0.05 are highlighted in bold.

| Regions | Effect size | p-value | Regions | Effect size | p-value |
|---|---|---|---|---|---|
| **Left-Lateral-Ventricle** | **-0.547** | **>0.001** | **lh_parstriangularis_volume** | **0.233** | **0.012** |
| **Left-Inf-Lat-Vent** | **-0.758** | **>0.001** | lh_pericalcarine_volume | 0.080 | 0.220 |
| Left-Cerebellum-White-Matter | 0.063 | 0.272 | lh_postcentral_volume | -0.123 | 0.117 |
| Left-Cerebellum-Cortex | -0.017 | 0.435 | **lh_posteriorcingulate_volume** | **0.435** | **>0.001** |
| **Left-Thalamus-Proper** | **0.282** | **0.003** | lh_precentral_volume | -0.029 | 0.391 |
| **Left-Caudate** | **0.321** | **0.001** | **lh_precuneus_volume** | **0.599** | **>0.001** |
| **Left-Putamen** | **0.287** | **0.003** | **lh_rostralanteriorcingulate_volume** | **0.289** | **0.003** |
| Left-Pallidum | -0.156 | 0.065 | **lh_rostralmiddlefrontal_volume** | **0.373** | **>0.001** |
| **3rd-Ventricle** | **-0.517** | **>0.001** | **lh_superiorfrontal_volume** | **0.362** | **>0.001** |
| 4th-Ventricle | -0.050 | 0.314 | **lh_superiorparietal_volume** | **0.303** | **0.002** |
| Brain-Stem | 0.126 | 0.112 | **lh_superiortemporal_volume** | **0.382** | **>0.001** |
| **Left-Hippocampus** | **0.790** | **>0.001** | **lh_supramarginal_volume** | **0.342** | **>0.001** |
| **Left-Amygdala** | **0.649** | **>0.001** | **lh_frontalpole_volume** | **0.211** | **0.020** |
| **CSF** | **-0.518** | **>0.001** | **lh_temporalpole_volume** | **0.477** | **>0.001** |
| **Left-Accumbens-area** | **0.325** | **0.001** | **lh_transversetemporal_volume** | **0.216** | **0.018** |
| Left-VentralDC | 0.138 | 0.091 | **lh_insula_volume** | **0.510** | **>0.001** |
| **Right-Lateral-Ventricle** | **-0.478** | **>0.001** | **rh_bankssts_volume** | **0.486** | **>0.001** |
| **Right-Inf-Lat-Vent** | **-0.768** | **>0.001** | rh_caudalanteriorcingulate_volume | 0.042 | 0.342 |
| Right-Cerebellum-White-Matter | 0.098 | 0.170 | rh_caudalmiddlefrontal_volume | 0.129 | 0.105 |
| Right-Cerebellum-Cortex | -0.024 | 0.409 | rh_cuneus_volume | -0.009 | 0.467 |
| Right-Thalamus-Proper | 0.117 | 0.129 | **rh_entorhinal_volume** | **0.603** | **>0.001** |
| **Right-Caudate** | **0.301** | **0.002** | **rh_fusiform_volume** | **0.506** | **>0.001** |
| **Right-Putamen** | **0.272** | **0.004** | **rh_inferiorparietal_volume** | **0.599** | **>0.001** |
| Right-Pallidum | -0.042 | 0.344 | **rh_inferiortemporal_volume** | **0.415** | **>0.001** |
| **Right-Hippocampus** | **0.635** | **>0.001** | **rh_isthmuscingulate_volume** | **0.370** | **>0.001** |
| **Right-Amygdala** | **0.651** | **>0.001** | **rh_lateraloccipital_volume** | **0.397** | **>0.001** |
| **Right-Accumbens-area** | **0.309** | **0.001** | **rh_lateralorbitofrontal_volume** | **0.305** | **0.002** |
| Right-VentralDC | 0.093 | 0.184 | **rh_lingual_volume** | **0.331** | **0.001** |
| CC_Posterior | 0.155 | 0.066 | **rh_medialorbitofrontal_volume** | **0.303** | **0.002** |
| CC_Mid_Posterior | 0.008 | 0.469 | **rh_middletemporal_volume** | **0.519** | **>0.001** |
| **CC_Central** | **0.258** | **0.006** | **rh_parahippocampal_volume** | **0.326** | **0.001** |
| **CC_Mid_Anterior** | **0.289** | **0.002** | rh_paracentral_volume | 0.114 | 0.135 |
| CC_Anterior | 0.041 | 0.345 | rh_parsopercularis_volume | 0.088 | 0.197 |
| **lh_bankssts_volume** | **0.394** | **0.000** | **rh_parsorbitalis_volume** | **0.325** | **0.001** |
| lh_caudalanteriorcingulate_volume | 0.037 | 0.361 | **rh_parstriangularis_volume** | **0.201** | **0.026** |
| **lh_caudalmiddlefrontal_volume** | **0.352** | **>0.001** | rh_pericalcarine_volume | 0.061 | 0.276 |
| lh_cuneus_volume | 0.037 | 0.361 | rh_postcentral_volume | -0.147 | 0.077 |
| **lh_entorhinal_volume** | **0.633** | **>0.001** | **rh_posteriorcingulate_volume** | **0.340** | **>0.001** |
| **lh_fusiform_volume** | **0.657** | **>0.001** | rh_precentral_volume | -0.022 | 0.417 |
| **lh_inferiorparietal_volume** | **0.526** | **>0.001** | **rh_precuneus_volume** | **0.570** | **>0.001** |
| **lh_inferiortemporal_volume** | **0.553** | **>0.001** | **rh_rostralanteriorcingulate_volume** | **0.240** | **0.010** |
| **lh_isthmuscingulate_volume** | **0.344** | **>0.001** | **rh_rostralmiddlefrontal_volume** | **0.304** | **0.002** |
| **lh_lateraloccipital_volume** | **0.322** | **0.001** | **rh_superiorfrontal_volume** | **0.330** | **0.001** |
| **lh_lateralorbitofrontal_volume** | **0.325** | **0.001** | **rh_superiorparietal_volume** | **0.410** | **>0.001** |
| **lh_lingual_volume** | **0.302** | **0.002** | **rh_superiortemporal_volume** | **0.350** | **>0.001** |
| **lh_medialorbitofrontal_volume** | **0.205** | **0.024** | **rh_supramarginal_volume** | **0.380** | **>0.001** |
| **lh_middletemporal_volume** | **0.543** | **>0.001** | rh_frontalpole_volume | 0.136 | 0.094 |
| **lh_parahippocampal_volume** | **0.501** | **>0.001** | **rh_temporalpole_volume** | **0.365** | **>0.001** |
| lh_paracentral_volume | -0.002 | 0.493 | rh_transversetemporal_volume | 0.141 | 0.085 |
| **lh_parsopercularis_volume** | **0.259** | **0.006** | **rh_insula_volume** | **0.426** | **>0.001** |
| **lh_parsorbitalis_volume** | **0.422** | **>0.001** | | | |

# 9. Univariate analysis – OASIS-1 dataset – HC vs AD

Supplementary Table 8 - Statistical significance measured by the Mann-Whitney U test and effect size measured by Cliff's delta absolute value based on the comparison of the reconstruction error for each brain region between the HC and the AD groups from the OASIS-1 dataset. The regions with p-value <= 0.05 are highlighted in bold.

| Regions | Effect size | p-value | Regions | Effect size | p-value |
|---|---|---|---|---|---|
| **Left-Lateral-Ventricle** | **-0.469** | **>0.001** | lh_parstriangularis_volume | 0.118 | 0.187 |
| **Left-Inf-Lat-Vent** | **-0.735** | **>0.001** | lh_pericalcarine_volume | -0.010 | 0.472 |
| **Left-Cerebellum-White-Matter** | **0.218** | **0.049** | **lh_postcentral_volume** | **0.362** | **0.003** |
| **Left-Cerebellum-Cortex** | **0.287** | **0.015** | **lh_posteriorcingulate_volume** | **0.359** | **0.003** |
| **Left-Thalamus-Proper** | **0.492** | **>0.001** | **lh_precentral_volume** | **0.330** | **0.006** |
| Left-Caudate | 0.040 | 0.382 | **lh_precuneus_volume** | **0.434** | **0.000** |
| Left-Putamen | 0.210 | 0.056 | lh_rostralanteriorcingulate_volume | 0.102 | 0.221 |
| Left-Pallidum | 0.030 | 0.413 | **lh_rostralmiddlefrontal_volume** | **0.337** | **0.005** |
| 3rd-Ventricle | -0.457 | **>0.001** | **lh_superiorfrontal_volume** | **0.358** | **0.003** |
| 4th-Ventricle | 0.006 | 0.484 | **lh_superiorparietal_volume** | **0.313** | **0.009** |
| **Brain-Stem** | **0.334** | **0.006** | **lh_superiortemporal_volume** | **0.502** | **>0.001** |
| **Left-Hippocampus** | **0.494** | **>0.001** | **lh_supramarginal_volume** | **0.502** | **>0.001** |
| **Left-Amygdala** | **0.481** | **>0.001** | lh_frontalpole_volume | 0.151 | 0.127 |
| **CSF** | **-0.350** | **0.004** | lh_temporalpole_volume | 0.032 | 0.405 |
| **Left-Accumbens-area** | **0.375** | **0.002** | **lh_transversetemporal_volume** | **0.343** | **0.005** |
| **Left-VentralDC** | **0.275** | **0.019** | **lh_insula_volume** | **0.313** | **0.009** |
| **Right-Lateral-Ventricle** | **-0.516** | **>0.001** | **rh_bankssts_volume** | **0.644** | **>0.001** |
| **Right-Inf-Lat-Vent** | **-0.780** | **>0.001** | rh_caudalanteriorcingulate_volume | 0.027 | 0.421 |
| Right-Cerebellum-White-Matter | 0.150 | 0.129 | **rh_caudalmiddlefrontal_volume** | **0.387** | **0.002** |
| **Right-Cerebellum-Cortex** | **0.227** | **0.043** | rh_cuneus_volume | 0.151 | 0.127 |
| **Right-Thalamus-Proper** | **0.521** | **>0.001** | **rh_entorhinal_volume** | **0.566** | **>0.001** |
| Right-Caudate | 0.002 | 0.496 | **rh_fusiform_volume** | **0.511** | **>0.001** |
| **Right-Putamen** | **0.339** | **0.005** | **rh_inferiorparietal_volume** | **0.462** | **>0.001** |
| Right-Pallidum | 0.007 | 0.480 | **rh_inferiortemporal_volume** | **0.572** | **>0.001** |
| **Right-Hippocampus** | **0.623** | **>0.001** | **rh_isthmuscingulate_volume** | **0.274** | **0.019** |
| **Right-Amygdala** | **0.541** | **>0.001** | **rh_lateraloccipital_volume** | **0.249** | **0.030** |
| **Right-Accumbens-area** | **0.424** | **0.001** | **rh_lateralorbitofrontal_volume** | **0.243** | **0.033** |
| **Right-VentralDC** | **0.354** | **0.004** | **rh_lingual_volume** | **0.313** | **0.009** |
| CC_Posterior | 0.183 | 0.084 | **rh_medialorbitofrontal_volume** | **0.312** | **0.009** |
| **CC_Mid_Posterior** | **0.496** | **>0.001** | **rh_middletemporal_volume** | **0.499** | **>0.001** |
| **CC_Central** | **0.503** | **>0.001** | **rh_parahippocampal_volume** | **0.430** | **0.001** |
| **CC_Mid_Anterior** | **0.432** | **0.001** | **rh_paracentral_volume** | **0.234** | **0.038** |
| **CC_Anterior** | **0.375** | **0.002** | **rh_parsopercularis_volume** | **0.297** | **0.012** |
| **lh_bankssts_volume** | **0.428** | **0.001** | **rh_parsorbitalis_volume** | **0.283** | **0.016** |
| lh_caudalanteriorcingulate_volume | 0.011 | 0.468 | **rh_parstriangularis_volume** | **0.283** | **0.016** |
| lh_caudalmiddlefrontal_volume | 0.113 | 0.198 | rh_pericalcarine_volume | 0.074 | 0.288 |
| lh_cuneus_volume | 0.200 | 0.065 | **rh_postcentral_volume** | **0.379** | **0.002** |
| **lh_entorhinal_volume** | **0.339** | **0.005** | rh_posteriorcingulate_volume | 0.183 | 0.084 |
| **lh_fusiform_volume** | **0.375** | **0.002** | **rh_precentral_volume** | **0.428** | **0.001** |
| **lh_inferiorparietal_volume** | **0.452** | **>0.001** | **rh_precuneus_volume** | **0.368** | **0.003** |
| **lh_inferiortemporal_volume** | **0.456** | **>0.001** | rh_rostralanteriorcingulate_volume | 0.114 | 0.195 |
| **lh_isthmuscingulate_volume** | **0.444** | **>0.001** | **rh_rostralmiddlefrontal_volume** | **0.425** | **0.001** |
| **lh_lateraloccipital_volume** | **0.314** | **0.009** | **rh_superiorfrontal_volume** | **0.425** | **0.001** |
| **lh_lateralorbitofrontal_volume** | **0.354** | **0.004** | **rh_superiorparietal_volume** | **0.272** | **0.020** |
| **lh_lingual_volume** | **0.238** | **0.036** | **rh_superiortemporal_volume** | **0.548** | **>0.001** |
| lh_medialorbitofrontal_volume | 0.064 | 0.316 | **rh_supramarginal_volume** | **0.519** | **>0.001** |
| **lh_middletemporal_volume** | **0.507** | **>0.001** | rh_frontalpole_volume | 0.126 | 0.171 |
| **lh_parahippocampal_volume** | **0.458** | **>0.001** | rh_temporalpole_volume | 0.007 | 0.480 |
| **lh_paracentral_volume** | **0.333** | **0.006** | **rh_transversetemporal_volume** | **0.413** | **0.001** |
| **lh_parsopercularis_volume** | **0.245** | **0.032** | **rh_insula_volume** | **0.292** | **0.014** |
| **lh_parsorbitalis_volume** | **0.270** | **0.021** | | | |

## 10. Univariate analysis –MIRIAD dataset – HC vs AD

Supplementary Table 9 - Statistical significance measured by the Mann-Whitney U test and effect size measured by Cliff's delta absolute value based on the comparison of the reconstruction error for each brain region between the HC and the AD groups from the MIRIAD dataset. The regions with p-value <= 0.05 are highlighted in bold.

| Regions | Effect size | p-value | Regions | Effect size | p-value |
|---|---|---|---|---|---|
| **Left-Lateral-Ventricle** | **-0.630** | **>0.001** | lh_parstriangularis_volume | 0.034 | 0.361 |
| **Left-Inf-Lat-Vent** | **-0.964** | **>0.001** | lh_pericalcarine_volume | -0.073 | 0.222 |
| Left-Cerebellum-White-Matter | 0.022 | 0.411 | **lh_postcentral_volume** | **0.394** | **>0.001** |
| **Left-Cerebellum-Cortex** | **0.396** | **>0.001** | **lh_posteriorcingulate_volume** | **0.545** | **>0.001** |
| **Left-Thalamus-Proper** | **0.470** | **>0.001** | **lh_precentral_volume** | **0.430** | **>0.001** |
| **Left-Caudate** | **0.338** | **>0.001** | **lh_precuneus_volume** | **0.841** | **>0.001** |
| **Left-Putamen** | **0.386** | **>0.001** | **lh_rostralanteriorcingulate_volume** | **0.440** | **>0.001** |
| Left-Pallidum | 0.092 | 0.169 | **lh_rostralmiddlefrontal_volume** | **0.836** | **>0.001** |
| **3rd-Ventricle** | **-0.385** | **>0.001** | **lh_superiorfrontal_volume** | **0.856** | **>0.001** |
| 4th-Ventricle | 0.059 | 0.269 | **lh_superiorparietal_volume** | **0.741** | **>0.001** |
| **Brain-Stem** | **0.334** | **>0.001** | **lh_superiortemporal_volume** | **0.753** | **>0.001** |
| **Left-Hippocampus** | **0.780** | **>0.001** | **lh_supramarginal_volume** | **0.788** | **>0.001** |
| **Left-Amygdala** | **0.878** | **>0.001** | **lh_frontalpole_volume** | **0.180** | **0.030** |
| **CSF** | **-0.673** | **>0.001** | **lh_temporalpole_volume** | **0.367** | **>0.001** |
| **Left-Accumbens-area** | **0.526** | **>0.001** | **lh_transversetemporal_volume** | **0.532** | **>0.001** |
| **Left-VentralDC** | **0.315** | **>0.001** | **lh_insula_volume** | **0.542** | **>0.001** |
| **Right-Lateral-Ventricle** | **-0.723** | **>0.001** | **rh_bankssts_volume** | **0.641** | **>0.001** |
| **Right-Inf-Lat-Vent** | **-0.932** | **>0.001** | **rh_caudalanteriorcingulate_volume** | **0.374** | **>0.001** |
| Right-Cerebellum-White-Matter | 0.131 | 0.086 | **rh_caudalmiddlefrontal_volume** | **0.471** | **>0.001** |
| **Right-Cerebellum-Cortex** | **0.418** | **>0.001** | **rh_cuneus_volume** | **0.430** | **>0.001** |
| **Right-Thalamus-Proper** | **0.416** | **>0.001** | **rh_entorhinal_volume** | **0.593** | **>0.001** |
| **Right-Caudate** | **0.183** | **0.028** | **rh_fusiform_volume** | **0.819** | **>0.001** |
| **Right-Putamen** | **0.406** | **>0.001** | **rh_inferiorparietal_volume** | **0.807** | **>0.001** |
| Right-Pallidum | 0.060 | 0.265 | **rh_inferiortemporal_volume** | **0.804** | **>0.001** |
| **Right-Hippocampus** | **0.802** | **>0.001** | **rh_isthmuscingulate_volume** | **0.712** | **>0.001** |
| **Right-Amygdala** | **0.877** | **>0.001** | **rh_lateraloccipital_volume** | **0.457** | **>0.001** |
| **Right-Accumbens-area** | **0.558** | **>0.001** | **rh_lateralorbitofrontal_volume** | **0.547** | **>0.001** |
| **Right-VentralDC** | **0.422** | **>0.001** | **rh_lingual_volume** | **0.396** | **>0.001** |
| CC_Posterior | 0.110 | 0.125 | **rh_medialorbitofrontal_volume** | **0.481** | **>0.001** |
| CC_Mid_Posterior | -0.067 | 0.244 | **rh_middletemporal_volume** | **0.825** | **>0.001** |
| **CC_Central** | **0.411** | **>0.001** | **rh_parahippocampal_volume** | **0.488** | **>0.001** |
| **CC_Mid_Anterior** | **0.500** | **>0.001** | rh_paracentral_volume | 0.150 | 0.058 |
| CC_Anterior | 0.083 | 0.193 | **rh_parsopercularis_volume** | **0.363** | **>0.001** |
| **lh_bankssts_volume** | **0.791** | **>0.001** | **rh_parsorbitalis_volume** | **0.232** | **0.008** |
| lh_caudalanteriorcingulate_volume | -0.095 | 0.162 | rh_parstriangularis_volume | -0.110 | 0.126 |
| **lh_caudalmiddlefrontal_volume** | **0.643** | **>0.001** | rh_pericalcarine_volume | 0.046 | 0.315 |
| lh_cuneus_volume | 0.156 | 0.052 | **rh_postcentral_volume** | **0.238** | **0.006** |
| **lh_entorhinal_volume** | **0.446** | **>0.001** | **rh_posteriorcingulate_volume** | **0.575** | **>0.001** |
| **lh_fusiform_volume** | **0.818** | **>0.001** | **rh_precentral_volume** | **0.424** | **>0.001** |
| **lh_inferiorparietal_volume** | **0.869** | **>0.001** | **rh_precuneus_volume** | **0.913** | **>0.001** |
| **lh_inferiortemporal_volume** | **0.884** | **>0.001** | **rh_rostralanteriorcingulate_volume** | **0.245** | **0.005** |
| **lh_isthmuscingulate_volume** | **0.604** | **>0.001** | **rh_rostralmiddlefrontal_volume** | **0.804** | **>0.001** |
| **lh_lateraloccipital_volume** | **0.628** | **>0.001** | **rh_superiorfrontal_volume** | **0.686** | **>0.001** |
| **lh_lateralorbitofrontal_volume** | **0.601** | **>0.001** | **rh_superiorparietal_volume** | **0.812** | **>0.001** |
| **lh_lingual_volume** | **0.304** | **0.001** | **rh_superiortemporal_volume** | **0.645** | **>0.001** |
| **lh_medialorbitofrontal_volume** | **0.535** | **>0.001** | **rh_supramarginal_volume** | **0.857** | **>0.001** |
| **lh_middletemporal_volume** | **0.882** | **>0.001** | **rh_frontalpole_volume** | **0.216** | **0.012** |
| lh_parahippocampal_volume | 0.140 | 0.072 | **rh_temporalpole_volume** | **0.437** | **>0.001** |
| **lh_paracentral_volume** | **0.381** | **>0.001** | **rh_transversetemporal_volume** | **0.393** | **>0.001** |
| **lh_parsopercularis_volume** | **0.314** | **0.001** | **rh_insula_volume** | **0.717** | **>0.001** |
| **lh_parsorbitalis_volume** | **0.333** | **>0.001** | | | |

# 11. Region importance – ADNI dataset – HC vs EMCI

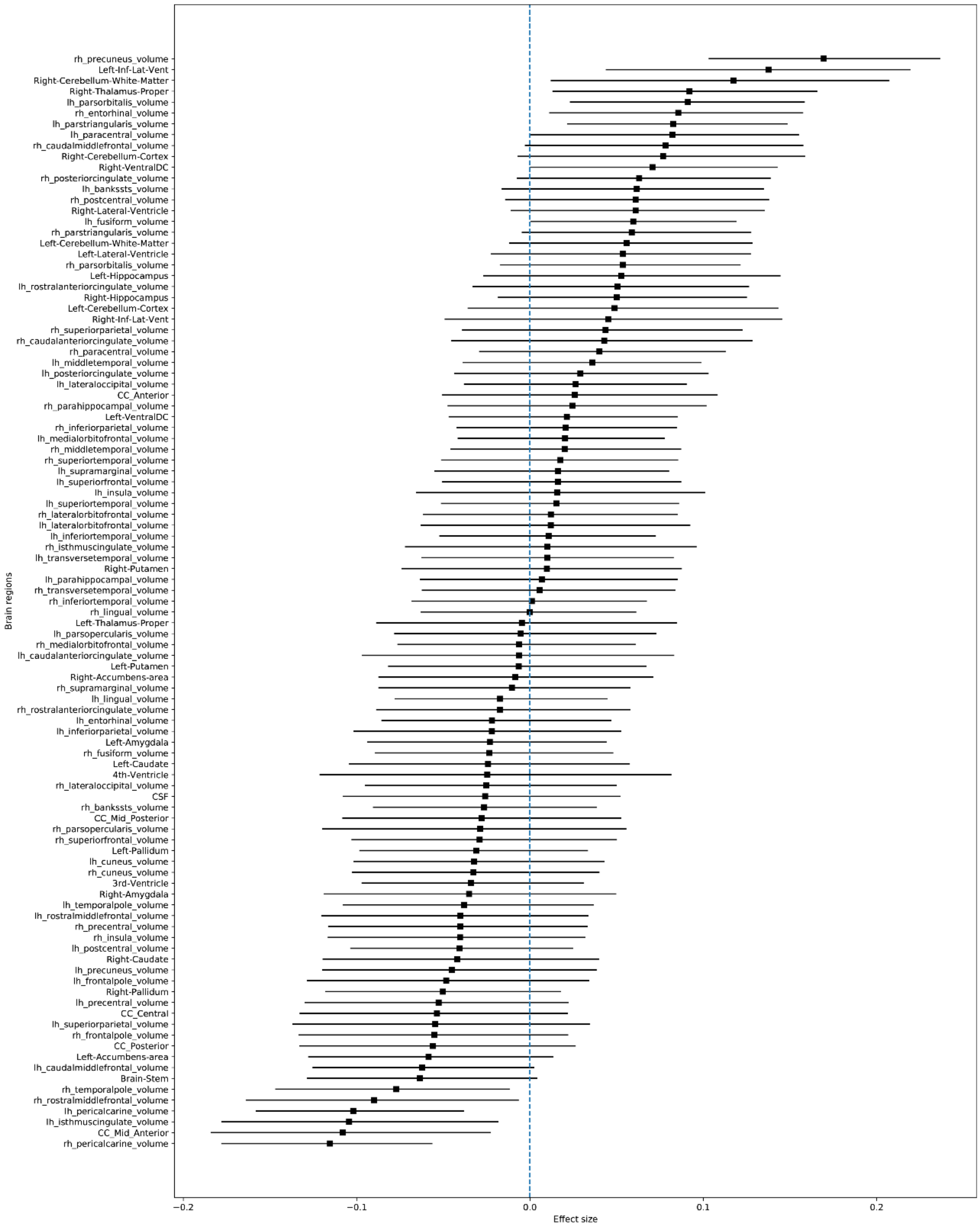


Supplementary Figure 1 - Regional deviations of the EMCI group from the ADNI dataset. The marker indicates the mean effect size between the HC and the EMCI groups. The horizontal bars indicate the 95% confidence interval calculated using the percentile method on the bootstrap analysis.

## 12. Region importance – ADNI dataset – HC vs LMCI

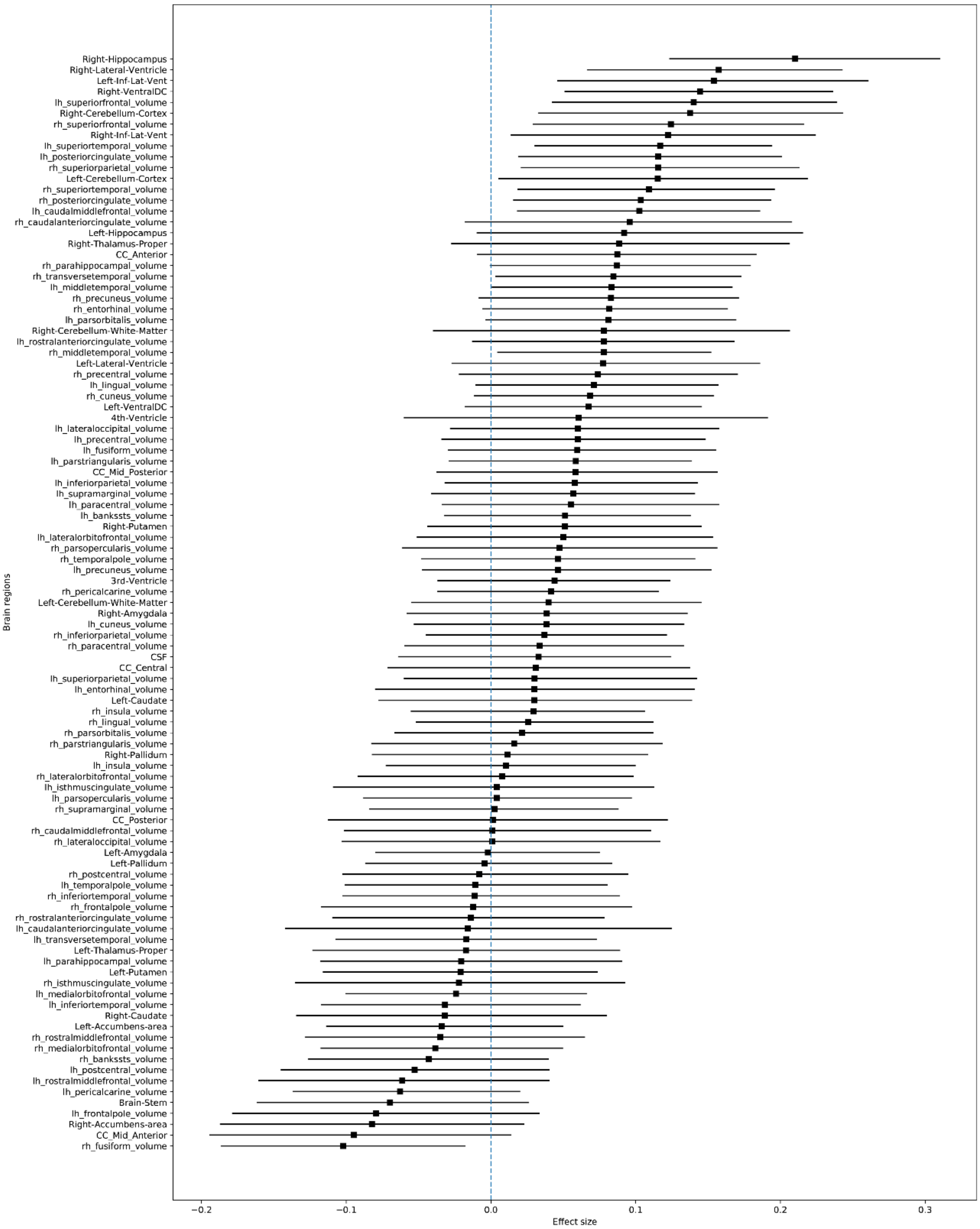


Supplementary Figure 2 – Regional deviations of the LMCI group from the ADNI dataset. The marker indicates the mean effect size between the HC and the LMCI groups. The horizontal bars indicate the 95% confidence interval calculated using the percentile method on the bootstrap analysis.

# 13. Region importance – ADNI dataset – HC vs AD

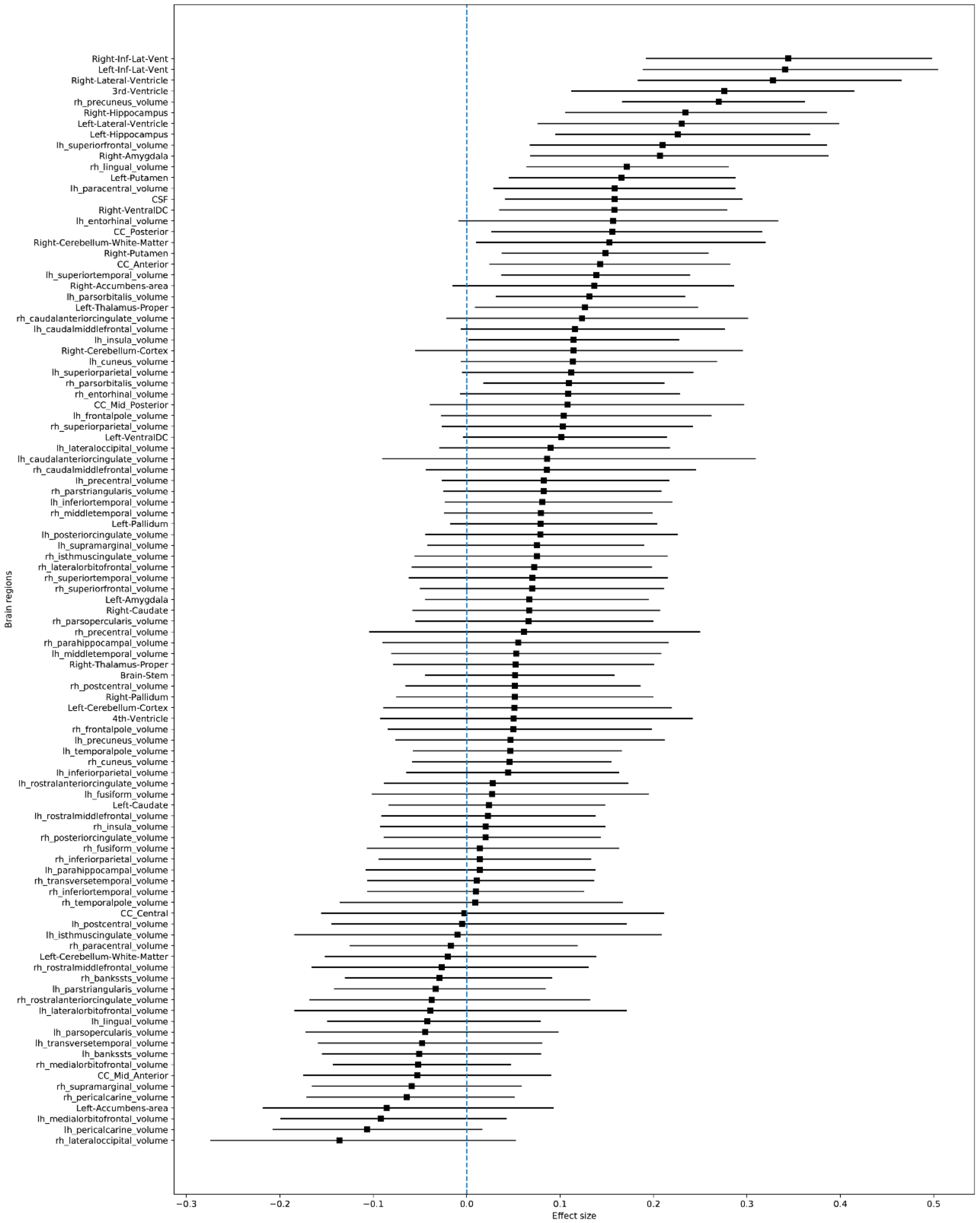


Supplementary Figure 3 – Regional deviations of the AD group from the ADNI dataset. The marker indicates the mean effect size between the HC and the AD groups. The horizontal bars indicate the 95% confidence interval calculated using the percentile method on the bootstrap analysis.

# 14. Region importance – AIBL dataset – HC vs MCI

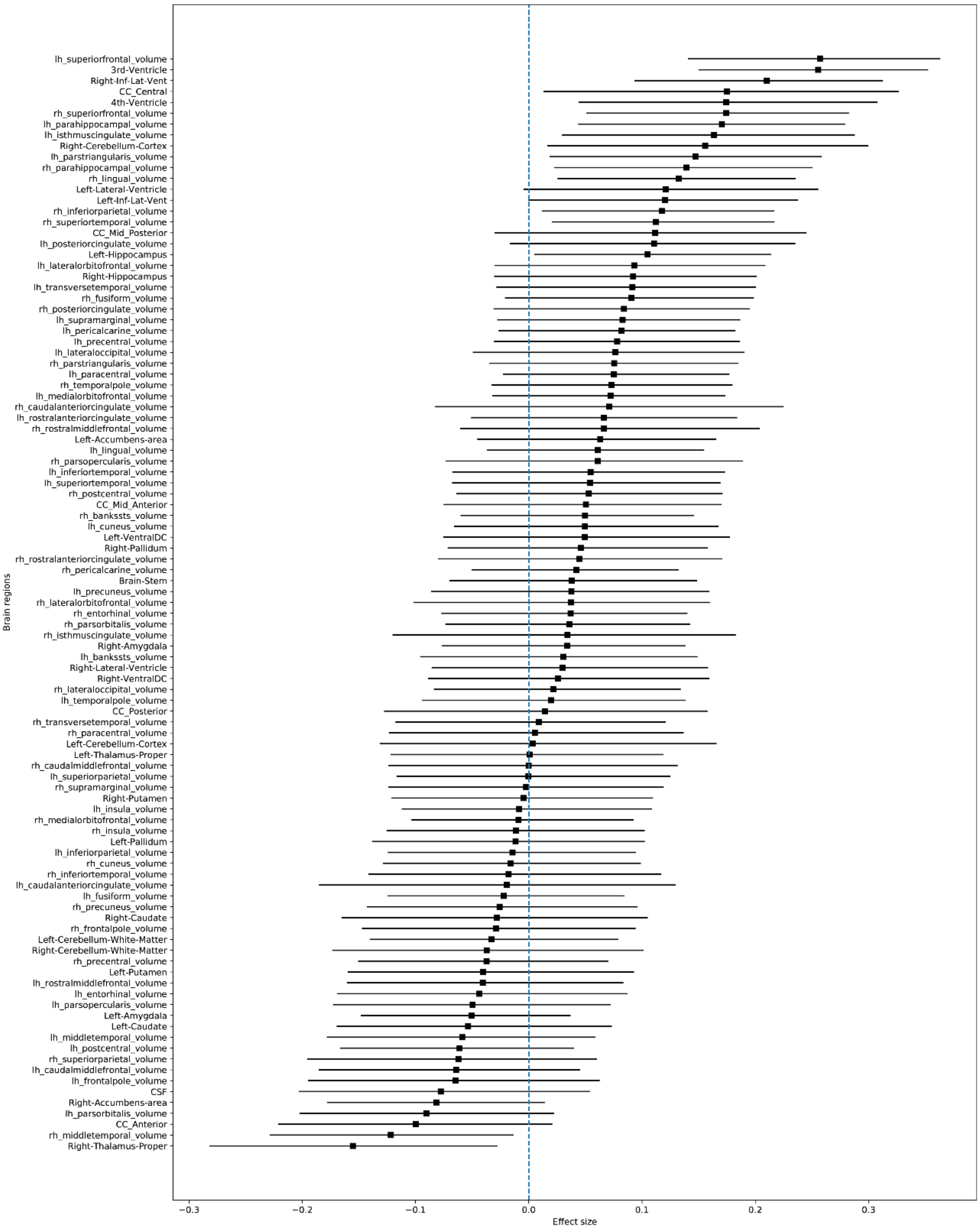


Supplementary Figure 4 – Regional deviations of the MCI group from the AIBL dataset. The marker indicates the mean effect size between the HC and the MCI groups. The horizontal bars indicate the 95% confidence interval calculated using the percentile method on the bootstrap analysis.

# 15. Region importance – AIBL dataset – HC vs AD

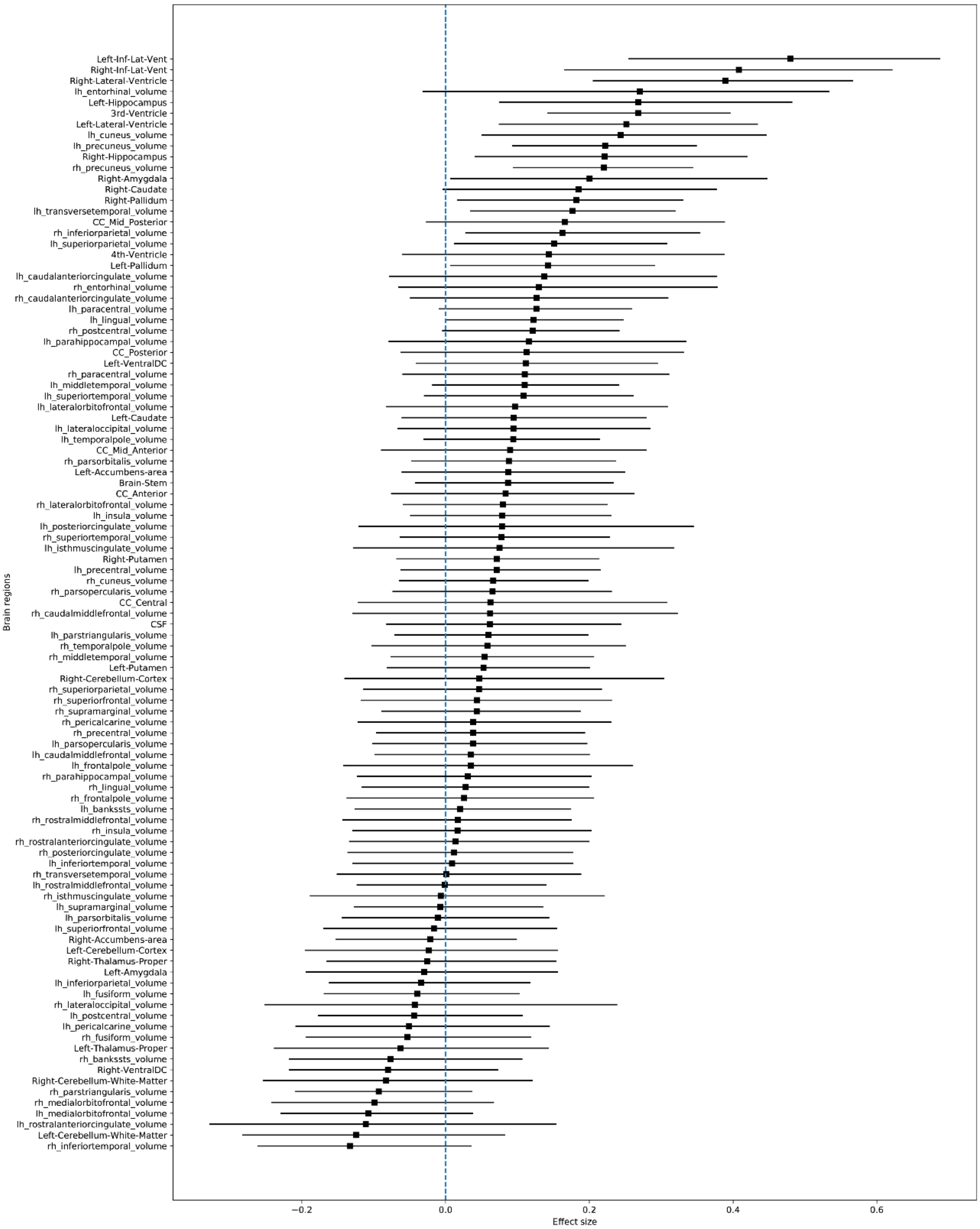


Supplementary Figure 5 – Regional deviations of the AD group from the AIBL dataset. The marker indicates the mean effect size between the HC and the AD groups. The horizontal bars indicate the 95% confidence interval calculated using the percentile method on the bootstrap analysis.

## 16. Region importance – ARWIBO dataset – HC vs MCI

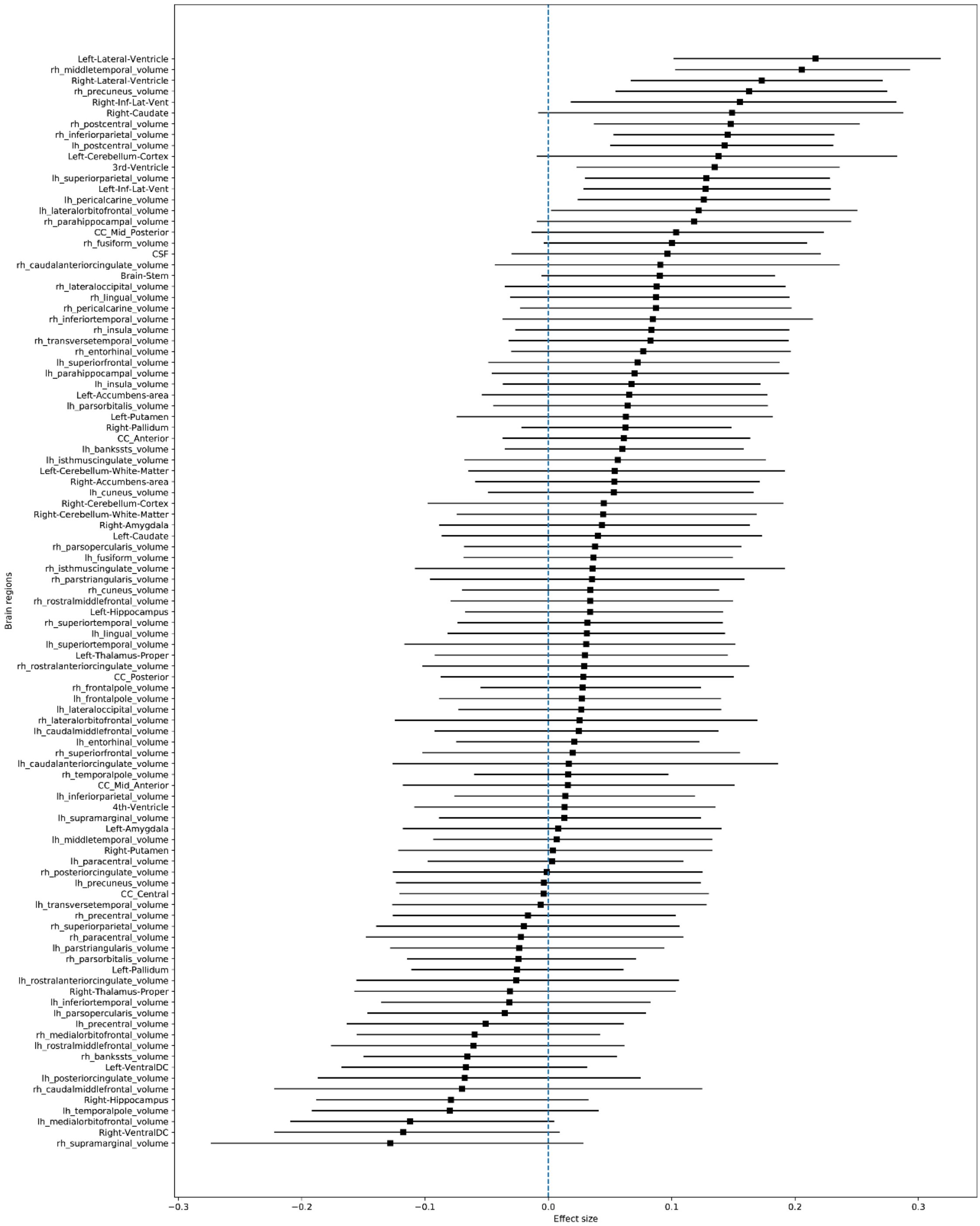


Supplementary Figure 6 - Regional deviations of the MCI group from the ARWIBO dataset. The marker indicates the mean effect size between the HC and the MCI groups. The horizontal bars indicate the 95% confidence interval calculated using the percentile method on the bootstrap analysis.

## 17. Region importance – ARWIBO dataset – HC vs AD

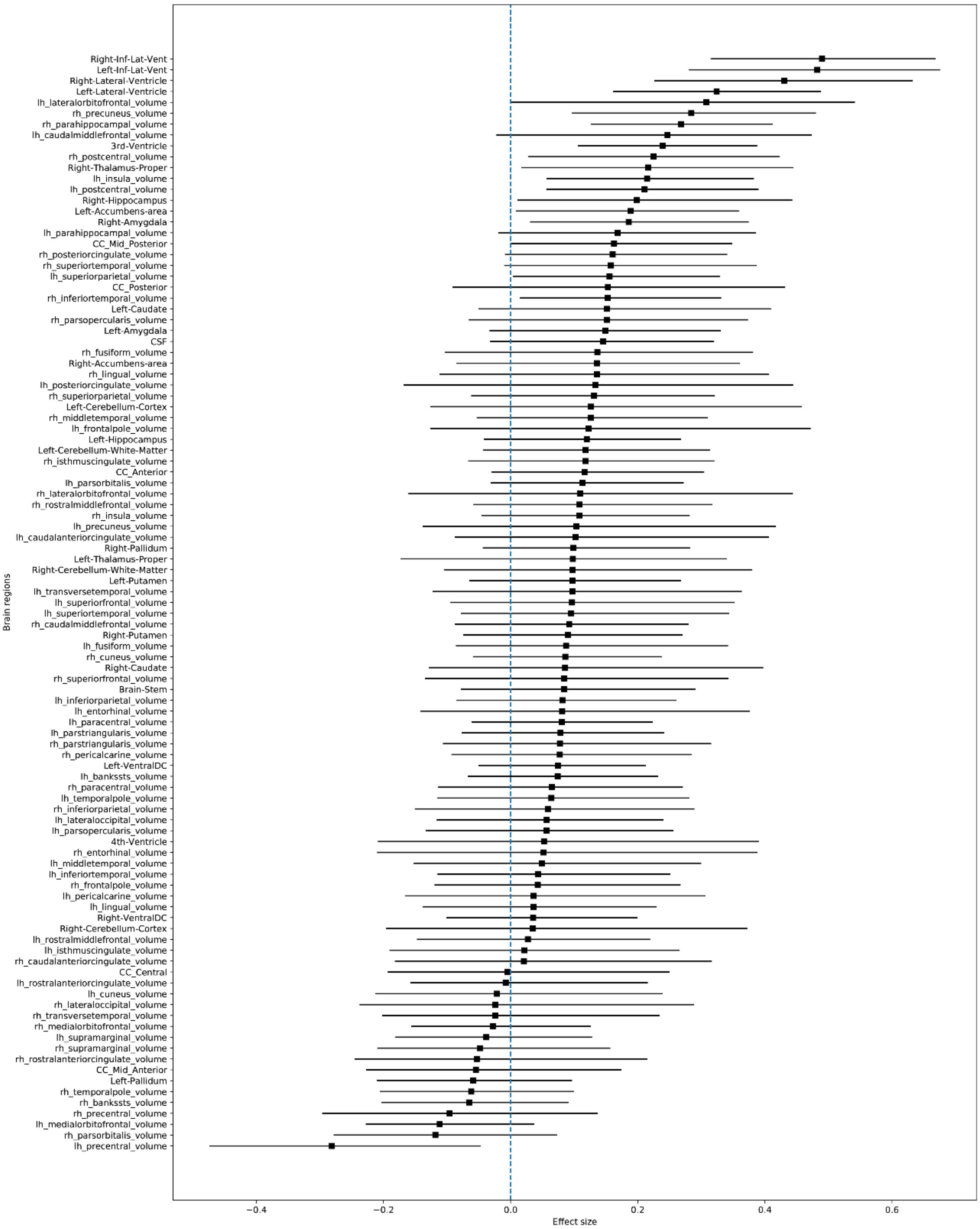


Supplementary Figure 7 – Regional deviations of the AD group from the ARWIBO dataset. The marker indicates the mean effect size between the HC and the AD groups. The horizontal bars indicate the 95% confidence interval calculated using the percentile method on the bootstrap analysis.

# 18. Region importance – OASIS-1 dataset – HC vs AD

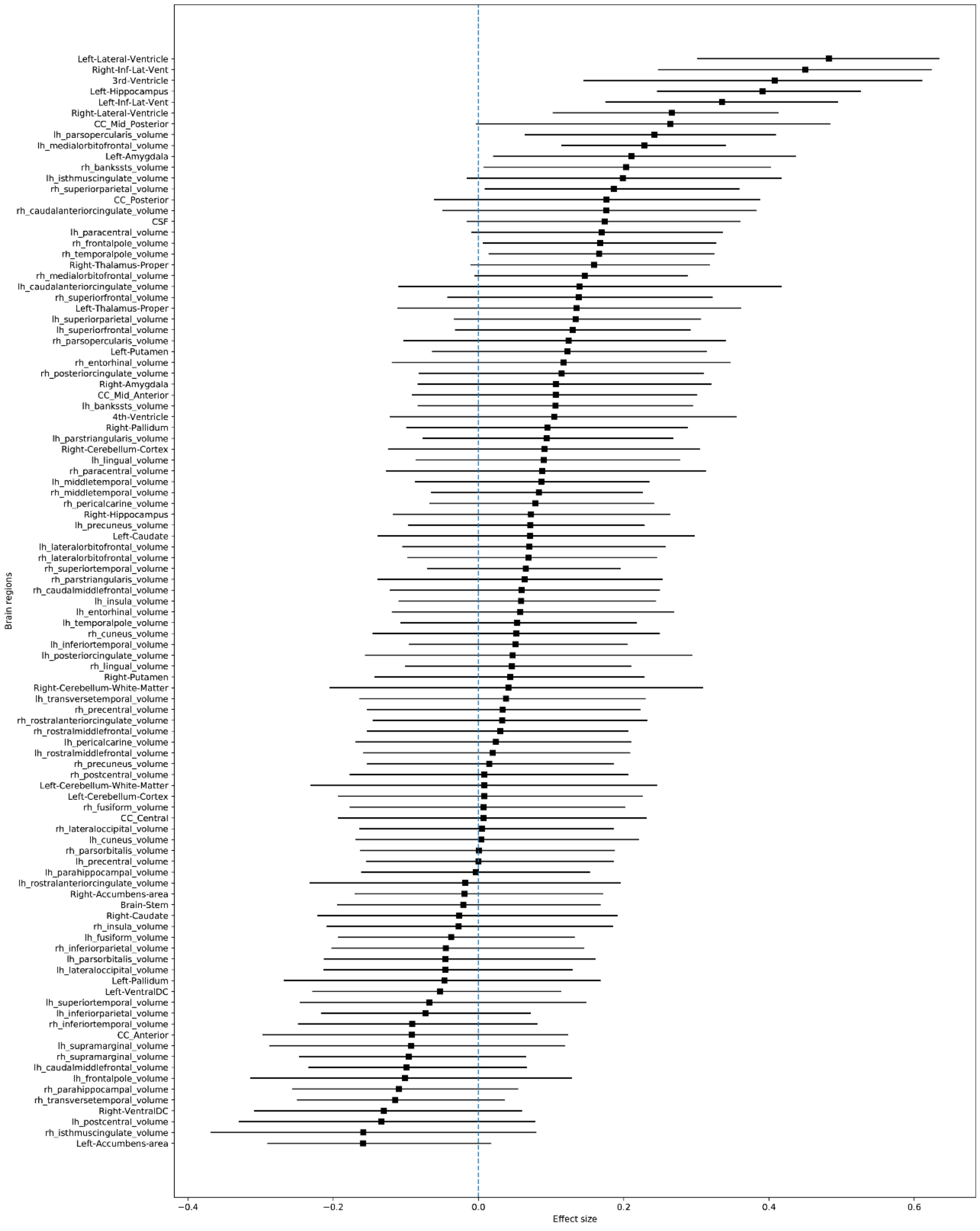


Supplementary Figure 8 - Regional deviations of the AD group from the OASIS-1 dataset. The marker indicates the mean effect size between the HC and the AD groups. The horizontal bars indicate the 95% confidence interval calculated using the percentile method on the bootstrap analysis.

# 19. Region importance – MIRIAD dataset – HC vs AD

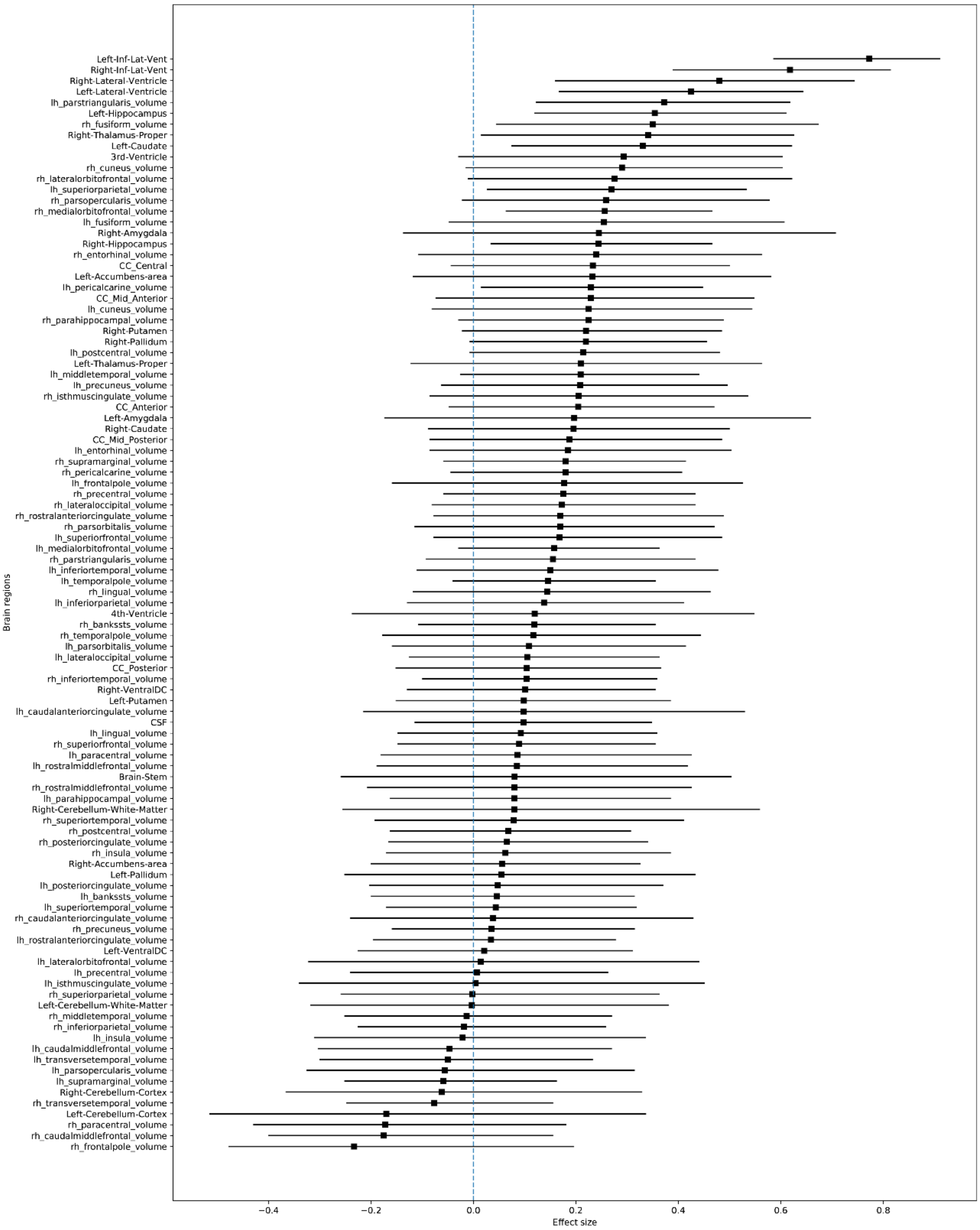


Supplementary Figure 9 - Regional deviations of the AD group from the MIRIAD dataset. The marker indicates the mean effect size between the HC and the AD groups. The horizontal bars indicate the 95% confidence interval calculated using the percentile method on the bootstrap analysis.

## 20. Comparison of deviation values for healthy controls and patients - confidence interval of the differences

When we analysed the confidence interval of the difference between groups in the observed deviation, we obtained that, for the ADNI dataset, the difference between HC and EMCI was in the range [-0.03, 0.00], the difference between HC and LMCI was in the range [-0.06, -0.03], the difference between HC and AD group was in the interval of [-0.16, -0.06], the difference between EMCI and LMCI was in the range [-0.03, -0.02], the difference between EMCI and AD was in the range [-0.14, -0.06], and the difference between LMCI and AD was in the range [-0.10, -0.03]. For the AIBL dataset, the difference between HC and MCI was in the range [-0.09, -0.05], the difference between HC and AD was in the range [-0.17, -0.07], the difference between MCI and AD was in the range [-0.09, 0.00]. For the ARWiBo dataset, the difference between HC and MCI was in the range [-0.08, -0.03], the difference between HC and AD was in the range [-0.24, -0.10], the difference between MCI and AD was in the range [-0.16, -0.06]. For the OASIS-1 dataset, the difference between HC and AD was in the range [-0.18, -0.33]. Finally, for the MIRIAD dataset, the difference between HC and AD was in the range [-0.16, -0.41]. In summary, the five independent datasets presented mean deviation scores significantly different between their groups, except the comparison between HC and EMCI in the ADNI dataset and the comparison between MCI and AD in the AIBL dataset.

## 21. Traditional machine learning classification - confidence interval of the differences

To identify significant differences between the performance of the normative models and traditional classifiers, we calculated the confidence interval (95% of confidence) of the difference in AUC between the two methods. For the ADNI dataset we found that when classifying HC and EMCI the difference was in the range [-0.28, -0.09], when classifying HC and LMCI the difference was in the range [-0.24, -0.04], and when classifying HC and AD the difference was in the range [-0.25, -0.12]. For the AIBL dataset, when classifying the HC and MCI the difference of performance was in the range [-0.17, 0.74], and when classifying HC and AD the difference was in the range [-0.29, -0.12]. For the ARWiBo dataset, when classifying the HC and MCI the difference of performance was in the range [-0.15, 0.12], and when classifying HC and AD the difference was in the range [-0.17, 0.00]. For the OASIS-1 dataset, when classifying HC and AD the difference was in the range [-0.25, 0.04]. Finally, For the MIRIAD dataset, when classifying HC and AD the difference was in the range [-0.15, 0.06]. In summary, the traditional classifiers were superior to the normative models when predicting the difference between the groups in the ADNI dataset and the difference between HC and AD for the AIBL dataset; in contrast the performance of the two approaches was comparable for all other comparisons.
